# Fast and quantitative compositional analysis of hybrid cellulose-based regenerated fibers using thermogravimetric analysis and chemometrics

**DOI:** 10.1007/s10570-021-03923-6

**Published:** 2021-05-28

**Authors:** Chamseddine Guizani, Mikaela Trogen, Hilda Zahra, Leena Pitkänen, Kaniz Moriam, Marja Rissanen, Mikko Mäkelä, Herbert Sixta, Michael Hummel

**Affiliations:** 1 Department of Bioproducts and Biosystems, Aalto University, P. O. Box 16300, 00076 Espoo, Finland; 2 VTT Technical Research Centre of Finland, Ltd, PO Box 1000, 02044 Espoo, Finland

**Keywords:** Ioncell^®^ technology, Compositional analysis, Man-made hybrid fibers, Biopolymers, Thermogravimetric analysis, Chemometrics

## Abstract

Cellulose can be dissolved with another biopolymer in a protic ionic liquid and spun into a bicomponent hybrid cellulose fiber using the Ioncell^®^ technology. Inside the hybrid fibers, the biopolymers are mixed at the nanoscale, and the second biopolymer provides the produced hybrid fiber new functional properties that can be fine-tuned by controlling its share in the fiber. In the present work, we present a fast and quantitative thermoanalytical method for the compositional analysis of man-made hybrid cellulose fibers by using thermogravimetric analysis (TGA) in combination with chemometrics. First, we incorporated 0–46 wt.% of lignin or chitosan in the hybrid fibers. Then, we analyzed their thermal decomposition behavior in a TGA device following a simple, one-hour thermal treatment protocol. With an analogy to spectroscopy, we show that the derivative thermogram can be used as a predictor in a multivariate regression model for determining the share of lignin or chitosan in the cellulose hybrid fibers. The method generated cross validation errors in the range 1.5–2.1 wt.% for lignin and chitosan. In addition, we discuss how the multivariate regression outperforms more common modeling methods such as those based on thermogram deconvolution or on linear superposition of reference thermograms. Moreover, we highlight the versatility of this thermoanalytical method—which could be applied to a wide range of composite materials, provided that their components can be thermally resolved—and illustrate it with an additional example on the measurement of polyester content in cellulose and polyester fiber blends. The method could predict the polyester content in the cellulose-polyester fiber blends with a cross validation error of 1.94 wt.% in the range of 0–100 wt.%. Finally, we give a list of recommendations on good experimental and modeling practices for the readers who want to extend the application of this thermoanalytical method to other composite materials.

**Supplementary Information:**

The online version contains supplementary material available at 10.1007/s10570-021-03923-6.

## Introduction

Ionic liquids (IL) can dissolve a wide range of biopolymers, including cellulose (Hermanutz et al. 2019), hemicelluloses, lignin (Pu et al. 2007), chitin and chitosan (Shamshina 2019), or natural composite matrices like wood (Ma et al. 2018a, b). Using the Ioncell^®^ technology (Sixta et al. 2015), we are able to dissolve mixtures of biopolymers in a protic ionic liquid and spin the solution into hybrid fibers. The obtained hybrid fibers are composed of two or more biopolymers forming nanoscale mixtures inside a micrometric fibrous matrix (Mikkilä et al. 2020; Zahra et al. 2020; Le et al. 2020; Trogen et al. 2021).

Hybrid fibers like cellulose-lignin (Bengtsson et al. 2019; Mikkilä et al. 2020; Le et al. 2020; Trogen et al. 2021), cellulose-chitosan (Zahra et al. 2020), cellulose-chitin (Ota et al. 2020) or cellulose-betulin (Makarov et al. 2018) have found applications in textiles (Ma et al. 2015) or as precursors for biobased carbon fibers (Byrne et al. 2016). The presence of a second biopolymer provides to the hybrid fiber new functional properties that can be fine-tuned by controlling the share of the biopolymer in the fiber.

The fiber composition is generally analyzed as a quality check and as a basis of process mass balance calculations after fiber spinning, washing, and drying. The macromolecular composition of the spun fibers can change in comparison to the initial biopolymers share used during the dissolution stage. Loss of biopolymers can occur during the dope filtration stage because of incomplete dissolution, or during the spinning operation due to diffusion and leaching of the biopolymers in the spin bath (Ma et al. 2015) (Mikkilä et al. 2020).

The compositional analysis of the hybrid fibers generally involves the use of wet-chemical methods for the hydrolysis and quantification of the biopolymers. Those methods are time consuming and require the use of several chemicals (more details in the materials and methods section). We therefore investigated the use of thermogravimetric analysis (TGA) as a faster alternative and green analytical method for the compositional analysis of the hybrid fibers (Koel and Kaljurand 2006; Armenta et al. 2008; Tobiszewski 2016).

The use of TGA has been applied to the compositional analysis of a wide range of composite materials. The method appears particularly efficient for measuring the contents of volatile additives, thermally stable organic fillers (e.g. carbon black) or inorganic fillers in composite materials (Brown 2005; Haines 2012). In most of these cases, the thermal analysis protocol is designed to separate the thermal decomposition stages of the different components. The resulting thermogram shows distinguished mass loss steps that correspond to the thermal decomposition stages of different components, and their contents can be directly read from the thermogram. Apart from few cases where such methods are applicable, they cannot be extrapolated to a wide range of composite (bio)materials. This is particularly true for lignocellulosic biomass and biocomposites, in which the thermal decomposition reactions of their structural biopolymers overlap (Yang et al. 2007; Sebio-Puñal et al. 2012; Yeo et al. 2019).

Several authors have tried to develop alternative methods for extracting compositional information from the TGA data of biomass. For instance, Cozzani et al. suggested a simple linear superposition of the thermograms of model biopolymers for quantifying their fractions in unknown biomass samples (Cozzani et al. 1997). Their method successfully predicted cellulose and lignin contents but showed limitations for predicting the hemicelluloses content. Carrier et al. proposed another method based on the deconvolution of the mass loss derivative curves (DTGs) into gaussian peaks, which areas are proportional to the biopolymers contents (Carrier et al. 2011). Although the authors obtained good correlations for holocellulose, α-cellulose and hemicelluloses (by difference), they were not able to accurately quantify the lignin content. Another variant of the deconvolution method was developed by Saldarriaga et al. (Saldarriaga et al. 2015), who suggested to quantify the different biopolymer contents (hemicellulose, cellulose, lignin) in a biomass by modeling their thermal decomposition reactions. Following this method, the biopolymer fractions and their pyrolysis kinetic parameters are identified from the deconvolution-fitting of the biomass thermogram according to the widely used kinetic model of parallel reactions (Grønli et al. 2002a; Anca-Couce et al. 2014). However, the authors assumed in their pyrolysis model that only lignin produces char, while cellulose and hemicelluloses fully volatilize. This assumption is of course highly disputable, since it is well known that cellulose and hemicellulose produce solid char during pyrolysis, with yields that are generally higher than 10 wt.% (Antal et al. 1998, 2000; Grønli et al. 1999; Lin et al. 2009; Zhou et al. 2017).

Besides the linear superposition and the deconvolution-based methods, combinations of thermoanalytical or spectroscopic methods with multivariate data analysis have been particularly successful for the compositional analysis of various materials (Risoluti et al. 2016; Acquah et al. 2017; García et al. 2017; Materazzi et al. 2017; Chauhan et al. 2020).

Here, we will discuss the combination of TGA and multivariate regression for the compositional analysis of hybrid cellulose-lignin and cellulose-chitosan man-made fibers. We will also discuss the versatility of this method and illustrate it with an additional example on the compositional analysis of cellulose-polyester textile fiber blends. We will also review and analyze some limitations of the linear superposition and deconvolution-based methods in comparison with the multivariate regression method, with a special emphasis on cases where the components in the composite material interact during the thermal decomposition. Finally, we will give some recommendations related to good experimental and modeling practices when using TGA and multivariate regression for compositional analysis.

## Material and methods

### Materials

Birch (Betula pendula) prehydrolysis kraft pulp (Mn = 57.5 kg/mol, Mw = 163.6 kg/mol, Enocell Speciality Cellulose, Finland) was received from Stora Enso Enocell Mill (Uimaharju, Finland). The cellulose was received as pulp sheets and ground to a fine powder in a Wiley mill before use. Organosolv (OS) beech lignin (Mn = 0.44 kg/mol, Mw = 2.23 kg/mol was received from the Lignocellulosic Biorefinery Pilot Plant, Fraunhofer CBP in Leuna, Germany. The lignin was used as received; only larger particles were crushed using a spoon before use. Chitosan powder (Mw = 30 kg/mol) was purchased from Glentham Life Science (UK). The ionic liquid (IL) 1,5-diazabicyclo[4.3.0]non-5-ene-1-ium acetate ([DBNH][OAc]) was synthesized from 1,5-diazabicyclo[4.3.0]non-5-ene (Fluorochem, UK) and acetic acid glacial (Merck, Germany), as described in (Michud et al. 2016).

## Biopolymer dissolution in IL and spinning of hybrid fibers

Cellulose pulp, mixtures of cellulose-lignin and cellulose-chitosan, with controlled mass proportions, were dissolved in [DBNH][OAc] inside a high shear kneader (80 °C, 90 min, 7 ± 3 mbar, mixing 30 rpm). The IL-biopolymers solutions were then filtered in a filter press unit (5 µm metal mesh pore size) and dry-jet wet spun using a piston spinning unit (Fourné Polymertechnik, Germany), as described in our previous articles (Zahra et al. 2020; Trogen et al. 2021). The spun fiber codes, their corresponding cellulose share and total biopolymers concentration during dissolution are summarized in Table 1. No 100% lignin or chitosan solutions could be spun into fibers because such solutions do not have the adequate viscoelastic properties needed for this particular IL-based dry-jet wet spinning (Zahra et al. 2020; Trogen et al. 2021).

**Table 1:** Experimental conditions during the preparation of hybrid Ioncell® fibers

Ioncell^®^ fiber	Cellulose pulp share during dissolution, wt.%	Biopolymers conc., wt.%	fiber code
Cellulose-lignin	100	13	Cellulose
	90	13	Cell90-BL10
	70	15	Cell70-BL30
	50	17	Cell50-BL50
Cellulose-chitosan	100	12	Cellulose
	90	12	Cell90-Ch10
	75	12	Cell75-Ch25
	50	12	Cell50-Ch50

## Fiber compositional analysis using reference methods

The sample preparation for carbohydrate and lignin analyses in the cellulose-lignin fibers were performed according to the NREL/TP-510-42,618 standard. The monosaccharides in the acid hydrolysis solutions were quantified using an ion chromatograph (ICS-3000 HPAEC-PAD), with a DionexTM CarboPacTM PA20 column (ThermoFisher Scientific, USA). Ultrapure water was used as eluent and after each analysis, the column was washed with NaOH solution, followed by ultrapure water for regeneration before the next injection. The acid-insoluble (Klason) lignin was determined by filtration and gravimetry, and the acid-soluble lignin (ASL) was measured using a Shimadzu UV 2550 spectrophotometer at 205 nm. The contents of the hemicelluloses, cellulose and lignin were calculated according to the Janson method (Janson 1972).

The chitosan contents in the cellulose-chitosan fibers were calculated from their nitrogen contents, which were measured using a Perkin Elmer 2400 CHNS/O elemental analyser. The C, H, and N (wt. %) contents were directly obtained from the measurement, while O (wt. %) was calculated by difference. Each sample was measured in duplicate. The acid hydrolysis protocol in the NREL/TP-510-42,618 standard did not work for the chitosan-cellulose fibers, as the chitosan could not be hydrolyzed using H_2_SO_4_. We tried a two-step hydrolysis protocol using H_2_SO_4_ and HCl to hydrolyze the cellulose and chitosan into their respective monosaccharides, but the hydrolyses were incomplete. We are still developing new wet-chemistry methods for the compositional analysis of those novel hybrid fibers and will therefore restrict our analysis to the chitosan content determined using elemental analysis.

## Thermogravimetric analysis

TGA was done in a Netzsch STA 449 F3 Jupiter & QMS 403 Aëolos Quadro thermal analyser. The fibers were heated from 40 to 600 °C at a heating rate of 10 °C/min under a flow of helium (70 mL/min). The sample mass was kept below 5 mg in order to minimize heat and mass transfer limitations (Narayan and Antal 1996). The calculations presented in Fig. S2 in the ESI, which are based on the references (Narayan and Antal 1996) (Lin et al. 2009) and (Richter and Rein 2018), suggest that the thermal lag in our experiments is only of few degrees. The measurements were repeated twice for each sample. From each thermogram, we extracted the following characteristic parameters that give a summary of the thermal reactivity of the sample:

- $T_{onset} , {^\circ }{\text{C}}$ temperature at a conversion level of 5%.
- $T_{peak} , {^\circ }{\text{C}}$: temperature at the maximum mass loss rate.
- $DTG_{peak} , \% /min$: maximum mass loss rate.
- $T_{{off{ }set}} , {^\circ }{\text{C}}$: temperature at a conversion level of 95%.
- $Y_{char} , \%$: final solid residue.

## Chemometric analysis of the thermograms

To derive compositional information from the TGA data, we used the samples thermograms as multivariate predictors in a Partial Least Squares Regression model (PLSR). The TGA data plotting, analysis and TGA-PLSR modelling were performed with Matlab (R2020b version, the Mathworks, Inc.). The TGA dataset used in this study is available from the corresponding author on a reasonable request.

The original thermograms (mass Vs temperature) were first preprocessed before the TGA-PLSR modelling. The thermograms were first adjusted on a dry sample basis by subtracting the mass loss occurring below 150 °C, which accounts for water removal during drying. Then, the thermograms were smoothed using a 20 points moving average filter and transformed into first derivative signals (DTG) using numerical differentiation. The preprocessed thermograms were then mean centered by subtracting the mean DTG signal of the DTG dataset.

**Fig. 1:**
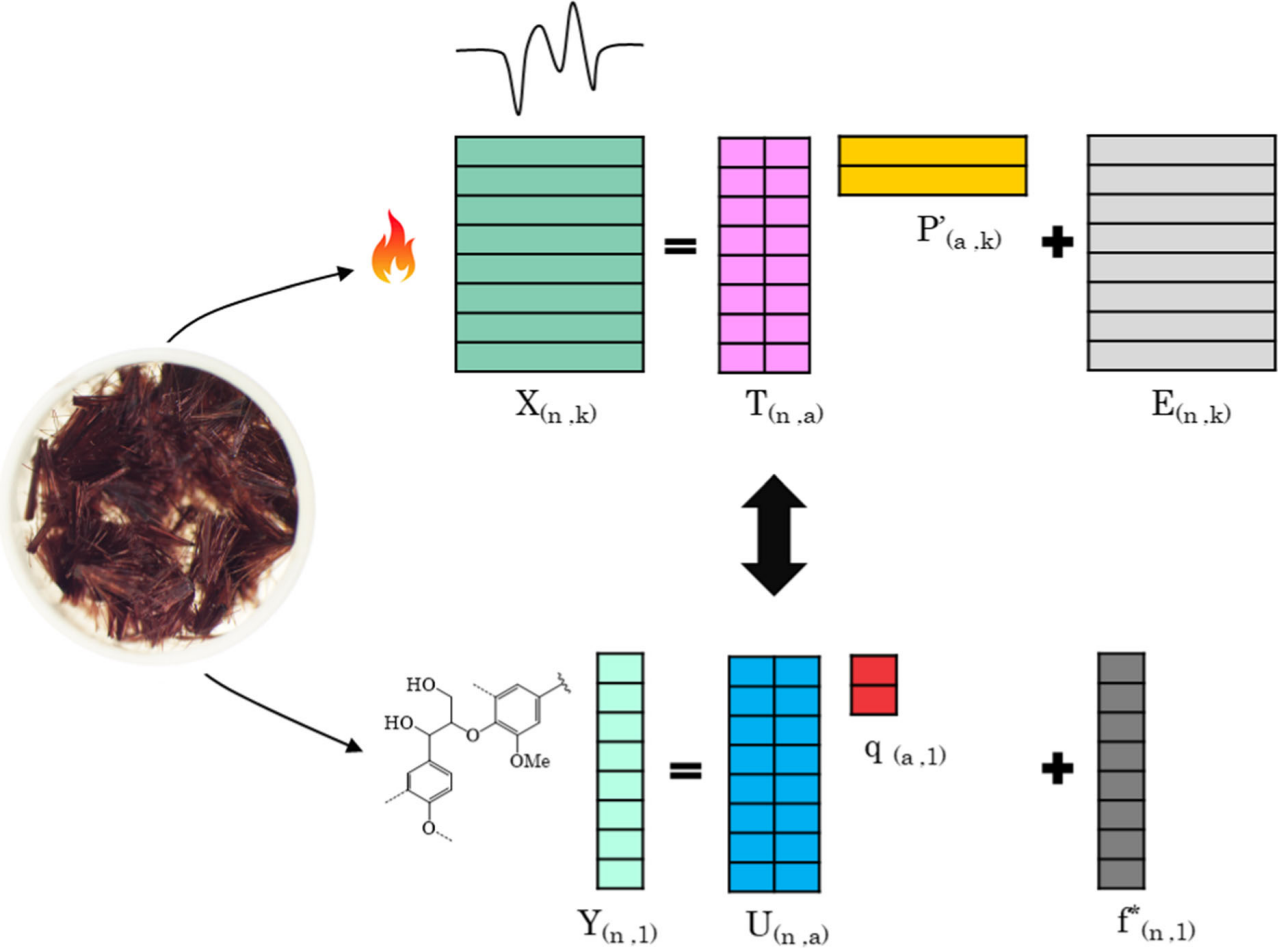
An illustration of the TGA-PLSR model principle for correlating the fiber TGA data to their biopolymer content. The Cell50BL50 fibers shown in the TGA crucible have distinct thermal signature and lignin content, which are correlated together through the PLSR

In general, PLSR is a method for relating an independent and dependent data set, here ${\varvec{X}}$ and ${\varvec{y}}$, by a linear multivariate regression model. The principle is based on calculating latent structures from both the ***X*** and ***y*** blocks by maximizing their covariance. In this way a regression model can be obtained even if the number of predictor variables exceeds the number of samples, which is a common situation in spectroscopy and thermogravimetric analysis. The procedure is illustrated in Fig. 1.

The preprocessed TGA data were first assembled into an ***X*** matrix, where the numbers of rows and columns respectively equalled the number of samples and variables in the pre-processed TGA data. Sample biopolymer contents were then organized in a column vector ${\varvec{y}}$ following the same order as in the ${\varvec{X}}$ matrix. The Non-Iterative Partial Least Squares (NIPALS) algorithm described by (Geladi and Kowalski 1986) was then used for building a regression model between ***X*** and ***y***.

In the NIPALS algorithm, the ${\varvec{X}}$ and ${\varvec{y}}$ blocks are decomposed into score and loading matrices, which for one **y** variable can be described as:

$${\varvec{X}} = \user2{TP^{\prime}} + {\varvec{E}}$$

$${\varvec{y}} = {\varvec{Uq}} + {\varvec{f}}*$$ where

- ${\varvec{T}}$ represent the TGA scores.
- $\user2{P^{\prime}}$ represent the TGA loading vectors.
- ${\varvec{U}}$ represent the concentration scores.
- ${\varvec{q}}$ are the concentration loadings.
- ${\varvec{E}}$ and ${\varvec{f}}^{\user2{*}}$ represent respectively the ${\varvec{X}}$ and ${ }{\varvec{y}}{ }$ blocks residuals after the projection onto a defined number of latent variables (LVs).

The scores ***T*** are orthogonal (***T***’***T*** is a diagonal matrix), which is enabled by the introduction of additional ***X*** block weights, ***W***, which are orthonormal (***W’W*** = ***I***). A mixed relation between ***X*** and ***y*** then exists through:

$${\varvec{y}} = \user2{Tq^{\prime}} + {\varvec{f}}$$

where ${\varvec{f}}$ denotes the difference between the observed **y** and predicted values of $\hat{\user2{y}}$ and is to be minimized. A regression vector ***b*** following the general regression:

$${\varvec{y}} = {\varvec{Xb}} + {\varvec{f}}$$

can also be determined as ${\varvec{b}} = {\varvec{W}}\left( {\user2{P^{\prime}W}} \right)^{ - 1} \user2{q^{\prime}}$**.**

Model validation and selection of the number of latent variables for a TGA-PLSR model were done according to a cross-validation procedure using the leave-one-out method (Brereton 2003). In this procedure, the number of latent variables was selected to minimize the root-mean square error of cross validation (${\varvec{RMSE}}_{{{\varvec{CV}}}}$):

$${\varvec{RMSE}}_{{{\varvec{CV}}}} = \sqrt {\frac{{\mathop \sum \nolimits_{{{\varvec{i}} = 1}}^{{\varvec{n}}} \left( {{\varvec{y}}_{{\varvec{i}}} - \hat{\user2{y}}_{{\varvec{i}}} } \right)^{2} }}{{\varvec{n}}}}$$

where ${\varvec{n}}$ denotes the number of samples in the data set. For more details on the PLSR method and algorithms, the reader is referred to (Helland 2001; Martens 2001; Wold et al. 2001a, b).

## Results and discussion

### Compositional analysis using reference methods

The Enocell cellulose pulp, which was the main component of the hybrid fibers was composed of 91.7% cellulose, 7.7% hemicelluloses, and 0.6% lignin. The beech lignin contained of 96.4% lignin, 0.3% cellulose, and 3.3% hemicelluloses. The compositions of the cellulose-lignin spun fibers were slightly different from the compositions we aimed for when we mixed the cellulose pulp and the beech lignin during the dissolution stage (see Table S1 in the ESI). It is likely that the water-soluble fractions of lignin and hemicelluloses did not coagulate during the spinning operation and leached out into the spin bath. It is also possible that part of these water-soluble fractions was removed in the washing step. The partial loss of hemicelluloses and lignin therefore increased the relative cellulose content in the spun fibers.

Similarly, we observed a lower chitosan content in the hybrid cellulose-chitosan fibers after spinning. Loss of undissolved chitosan chains during filtration and leaching of the chitosan short chains in the spinning bath during the spinning or washing steps can explain those deviations. The measured chitosan content in the different cellulose-chitosan fibers is given in Table S2 in the ESI).

## Thermogravimetric analysis

### Cellulose-lignin samples

**Fig. 2:**
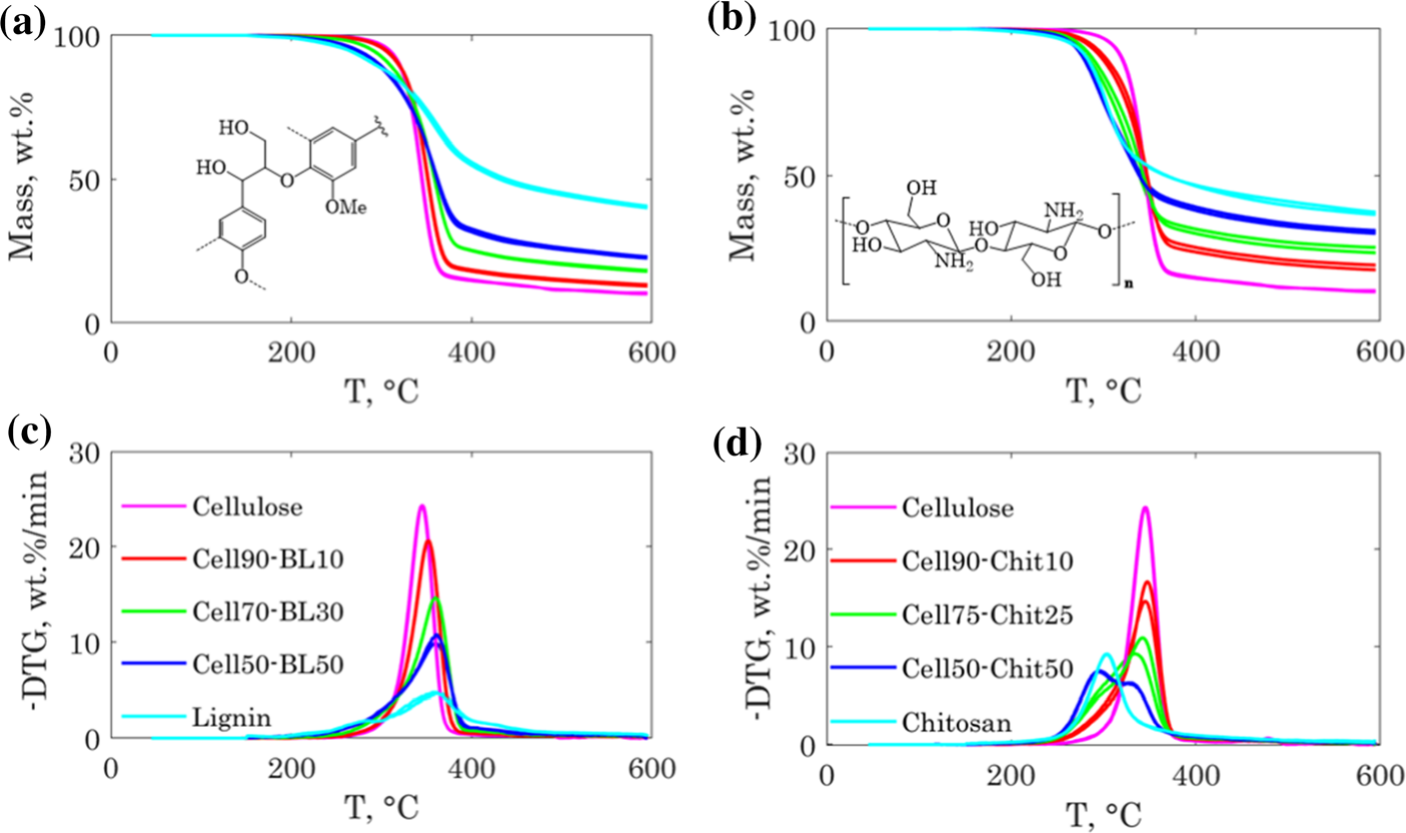
TGA and DTG curves of the cellulose-lignin samples (**a** and **c**) and cellulose-chitosan samples (**b** and **d**)

The TGA and the DTG curves of the cellulose-lignin samples are shown in Fig. 2 (a and c). The TGA curves of the two repeatability tests for each sample are almost superimposed. The root mean square deviation between two repeatability tests was less than 1 wt.% for all the samples (see Fig. S3 in the ESI). The thermograms and their derivatives showed clear changes when increasing the share of lignin in the hybrid fiber. The characteristic parameters of the thermograms are summarized in Table 2. Plots of the TGA characteristic parameters are also shown in Fig. S4 in the ESI.

**Table 2:** TGA characteristic parameters for the cellulose-lignin and cellulose-chitosan samples

	Sample	$T_{onset}$	±	$T_{peak}$	±	$DTG_{peak}$	±	$Y_{char}$	±	$T_{{off{ }set}}$	±
Cellulose-lignin	Cellulose	306.8	0.4	346.3	0.4	25.8	0.0	10.3	0.2	404.3	11.0
	Cell90-BL10	301.8	1.1	353.3	0.4	21.8	0.1	13.1	0.4	417.8	0.4
	Cell70-BL30	284.3	0.4	360.8	0.4	15.2	0.0	18.2	0.2	449.3	1.1
	Cell50-BL50	261.8	3.9	361.8	1.1	10.6	0.7	22.9	0.3	479.3	4.6
	Beech lignin	241.5	3.5	361.8	5.3	4.8	0.0	40.4	0.5	529.3	1.8
Cellulose-chitosan	Cellulose	306.8	0.4	346.3	0.4	25.8	0.0	10.3	0.2	404.3	11.0
	Cell90-Chit10	279.8	3.2	347.5	1.4	16.6	1.7	18.5	1.1	437.8	0.4
	Cell75-Chit25	265.3	4.6	339.0	6.4	10.4	1.3	24.5	1.3	447.3	0.4
	Cell50-Chit50	261.0	1.4	294.3	0.4	7.7	0.0	30.7	0.7	474.8	2.5
	Chitosan	251.3	1.8	304.5	0.0	9.7	0.1	37.0	0.6	505.0	9.9

$T_{onset}$ and $DTG_{peak}$ decreased when increasing the share of lignin in the fiber, while $T_{peak}$, $T_{offset}$, and $Y_{char}$, increased accordingly. The changes in the thermogram profile can be explained by the larger thermal degradation window of lignin and its higher char yield compared to cellulose (Yang et al. 2007) (Yang et al. 2006), which decomposes in a narrower temperature range, as shown for the pure cellulose fiber.

The $T_{peak}$ shifted towards higher temperatures when increasing the share of lignin in the fiber. This shift suggested possible interactions between cellulose and lignin during the pyrolysis reaction. This was likely the case, since the two biopolymers are mixed at the nanoscale level. However, it is not clear if there were chemical interactions behind the observed shifts, or if it was related to other physical phenomena. For instance, lignin softening (Shrestha et al. 2017; Han et al. 2019) might cause delays in the cellulose pyrolysis mass loss because of mass transfer limitations. A simple way to investigate the presence of interactions between the biopolymers during the thermal decomposition of the hybrid fibers is to compare the measured hybrid fibers thermograms with those calculated using the thermograms of lignin and cellulose following a linear superposition law. Deviations from the superposition law indicate the presence of interactions. We will discuss this point more in detail later in this paper.

### Cellulose-chitosan samples

The thermograms of the chitosan and cellulose-chitosan fibers, as well as their derivative signals are shown in Fig. 2b and d. For the cellulose-chitosan samples, the measurement repeatability was slightly worse than for the cellulose-lignin samples. The root-mean square deviation between two repeatability tests varied between 0.25 and 1.24 wt.% (see Fig. S5 in the ESI). The thermograms showed clear differences when increasing the share of chitosan in the hybrid fibers. The characteristic parameters of the thermograms are summarized in Table 2**.** The thermograms characteristic parameters as a function of the chitosan share are also shown in Fig. S6 in the ESI.

Chitosan and cellulose have very different thermal behaviour despite their structural similarity. The presence of amine and acetamide groups on the backbone structure of the chitosan chains favours the formation of char during pyrolysis through condensation reactions (López et al. 2008; Zeng et al. 2011). The char yield was 37 wt.% for chitosan, compared to 10.7 wt.%% for cellulose. Chitosan has also a lower thermal stability than cellulose and start to decompose at a lower ${T}_{onset}$. The hybrid fibers showed consequently a lower thermal stability and a higher char yield as the share of chitosan increased in the fibers. However, those changes were not linear since the char yields were not additive and the cellulose maximum decomposition peak shifted to lower temperatures. The nanoscale mixing of the cellulose and chitosan chains promoted interactions between chitosan and cellulose during pyrolysis, which altered significantly the thermograms and increased the char yield (Zahra et al. 2020).

## Composition analysis using the TGA-PLSR method

**Fig. 3:**
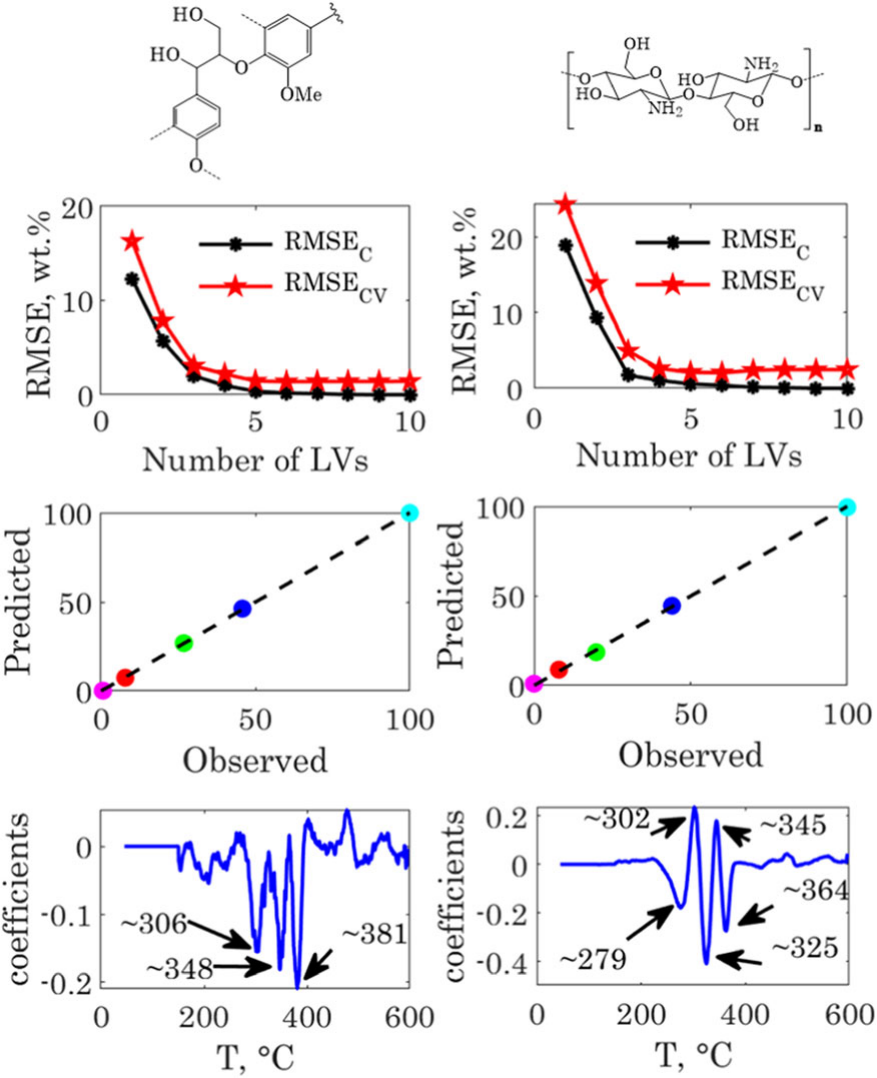
TGA-PLSR results for the cellulose-lignin (left) and cellulose chitosan samples (right). From top to down: RMSEs, predicted Vs observed content and model regression vector

The preprocessing steps of the cellulose-lignin and cellulose-chitosan samples thermograms are illustrated respectively in Fig. S7 and Fig. S9 in the ESI. The TGA-PLSR results for the determination of the lignin and chitosan contents, respectively in the cellulose-lignin and cellulose-chitosan samples are shown in Fig. 3. We will now discuss those results for each group of samples separately.

### The cellulose-lignin samples

The top-left graph in Fig. 3 shows the variation of the root mean square errors of calibration (RMSEC) and cross validation (RMSECV) with the number of LVs included in the model. The RMSECV was used for choosing the number of LVs for TGA-PLSR model. As illustrated in Fig. 3, the RMSECV decreased significantly when including five LVs in model, and then remained almost constant. Adding more than five LVs would not hence improve the model quality. From the analysis of the different LVs score and loading plots, (see Fig. S8 in the ESI), the five first loadings show a clear structure and appear to contribute, with different extents, in modeling the data variability. Therefore, we chose to include five LVs in the TGA-PLSR model for predicting the lignin content in the cellulose-lignin samples.

With five LVs, the explained variance in the **X** and **y** block exceeded 99%. The RMSECV was 1.46 wt.% and the coefficient of determination was higher than 0.99. The model prediction performance was satisfactory and shows that this method can be used for predicting the lignin content in the regenerated fibers with a good accuracy.

The regression vector, shown in the bottom-left graph of Fig. 3, showed three main peaks corresponding to the most important regression coefficients. Those coefficients can be linked to the temperatures for which there are significant differences in the thermal behavior of the samples. The first two coefficients at 306 °C and 348 °C corresponded to the onset and DTG peak temperature of the cellulose fiber. The last one, at 381 °C, was close to the temperature for which the mass loss rate of cellulose approached zero. Those coefficients were all negative since they are correlated with the cellulose content. Positive peaks appeared at higher temperatures and were positively correlated to the lignin content, which had a broader decomposition range. This relationship between the regression vector coefficients and characteristic temperatures of cellulose and lignin decompositions illustrates very well how the TGA-PLSR works for coupling the thermal behavior of a biopolymer to its content in the hybrid fiber.

The score and loading plots depicted in Fig. S8 in the ESI show that the first LV accounted for nearly 81% of the ***X*** block variance and separated the samples as a function of their lignin content. Samples with the lowest lignin contents had the lowest scores. The corresponding loading vector was meaningful to this regard, since it showed a main negative peak with a minimum corresponding to the decomposition peak temperature of the cellulose fiber (around 345 ºC) and gave negative scores to the samples with a high cellulose content. The second LV accounted for nearly 17% of the ***X*** block variability and separated the extreme samples (the cellulose fiber and the beech lignin) from the hybrid fibers. The loading vector showed also two distinguished and opposite peaks. The first negative at 339ºC, and the second positive one at 365ºC. Though accounting for a small part of the data variability, the rest of the LVs loading vectors showed a clear structure which might be related to the maximum decomposition peak shift when increasing the lignin content. The reconstructed thermograms using the model scores and loadings are shown in Fig. S9 in the ESI, together with the experimental ones. This figure illustrates how powerful the TGA-PLSR model can be in reconstructing accurately the original thermograms. The main results from the TGA-PLSR models for the quantification of the three biopolymers in the cellulose-lignin samples are finally summarized in Table 3. They were all satisfactory when evaluating their respective RMSECVs, which were in the same order of magnitude as the experimental errors. The respective coefficients of determination were also all close to one.

**Table 3:** TGA-PLSR results for the cellulose-lignin and cellulose-chitosan samples

	Biopolymer	LVs	$Var_{X}$, %	$Var_{y}$, %	$RMSE_{C}$, wt.%	$RMSE_{CV}$, wt.%	R^2^	Range, wt.%
Cellulose-lignin	Lignin	5	99.9	99.96	0.37	1.46	0.999	0.6–96.4
	Cellulose	5	99.9	99.96	0.35	1.36	0.999	0.3–91.9
	Hemicelluloses	5	99.9	99.96	0.13	0.21	0.999	3.3–7.5
Cellulose-chitosan	Chitosan	4	99.9	99.9	1.05	2.12	0.999	0–100

### The cellulose-chitosan samples

The model RMSECV decreased when adding up to five LVs in the model, then became almost constant. However, when visualizing the fifth LV loading vector, this later appeared highly noisy and did not describe meaningful information. Therefore, we decided to exclude it from the TGA-PLSR model. The four LVs model explained more than 99.9% of the variability in the **X** and **y** blocks and predicted remarkably well the chitosan content. The model RMSECV was 2.1 wt.% within a range of 0–100 wt.% of chitosan and the coefficient of determination was higher than 0.99. It is interesting to note that the first LV explained more than 97% of the X block variability (see Fig. S10 in the ESI), but only about 72% of the y block variability. The first LV loading vector and score plots indicated that it separated the samples as a function of their chitosan content. The loading vector showed two main peaks, a positive one at ~ 298 °C and a more pronounced negative one at ~ 347 °C. Those two temperatures corresponded with the peak decomposition temperatures of chitosan and cellulose, respectively.

The regression vector, shown in the bottom-left graph of Fig. 3, has five main peaks corresponding to the most important regression coefficients. These peaks can be related to the thermal decomposition temperatures of cellulose and chitosan, as previously discussed for the cellulose-lignin samples. The reconstructed thermograms using the model scores and loadings are shown together with the experimental ones in Fig. S11 in the ESI. They are very well reproduced by the model. We calculated that the maximum difference between the measured and modelled DTG curves was less than 0.2 wt.%/min for all the samples and through the whole temperature range. The fact that more LVs were needed to predict the chitosan content in the samples is most likely due the nonlinear effects generated by the synergy between cellulose and chitosan during the pyrolysis reaction. As the chitosan catalyzed the dehydration and char formation during cellulose pyrolysis, the observed cellulose and chitosan peak intensity and position did not evolve linearly with their respective shares in the hybrid samples. This is a very important point which can explain how the TGA-PLSR models can succeed where the classical modeling approaches based on superposition or deconvolution fail. A comparison of those modeling approaches will be discussed in the next section.

## Soft statistical models or hard physical models?

In this section, we will compare the TGA-PLSR modelling approach, which we discussed so far, to more classical approaches based on linear superposition or deconvolution. To do so, we propose to test the method of Saldarriaga et al. (Saldarriaga et al. 2015) who suggested identifying the biopolymer contents (hemicellulose, cellulose, lignin) in biomass by modelling the kinetics of their respective pyrolysis reactions.

The methodology is based on the well-known three parallel reaction model (Grønli et al. 2002b). Following this model, cellulose, hemicellulose, and lignin undergo parallel pyrolysis reactions independently. The sum of the released volatiles corresponds to the total mass loss observed during a TGA experiment. The pyrolysis dynamic of a biopolymer “i” is described by a first-order reaction:

$$\frac{{dm_{i} }}{dt} = - A_{i} e^{{ - \frac{{E_{i} }}{RT}}} m_{i}$$

$$m_{i} \left( 0 \right) = {\upnu }_{i}$$

where ${\upnu }_{i}$ is the weigh fraction of a biopolymer “i” in the fiber sample.

The dynamics of the fiber mass loss is hence described by:

$$\frac{{dm_{f} }}{dt} = \mathop \sum \limits_{i = 1}^{3} c_{i} \frac{{dm_{i} }}{dt}$$

where $m_{f}$ is the fiber mass and $c_{i}$ is the fraction of volatiles released during the pyrolysis of the biopolymer “i”. A set of kinetic parameters and volatile fractions for each of the biopolymers can be identified by minimizing the following objective function using a non-linear least square solver:

$$OF = \mathop \sum \limits_{k = 1}^{N} \frac{{\left( {m_{k} - \hat{m}_{k} } \right)^{2} }}{N}$$

where $m_{k}$ and $\hat{m}_{k}$ are the respective experimental and modelled mass for the kth point and N is the number of experimental points in a thermogram.

The fit quality is assessed through the following parameter:

$$Fit \% = \frac{{\sqrt {\mathop \sum \nolimits_{i = 1}^{N} \left( {m_{i} - \hat{m}_{i} } \right)^{2} } }}{N}$$

**Fig. 4:**
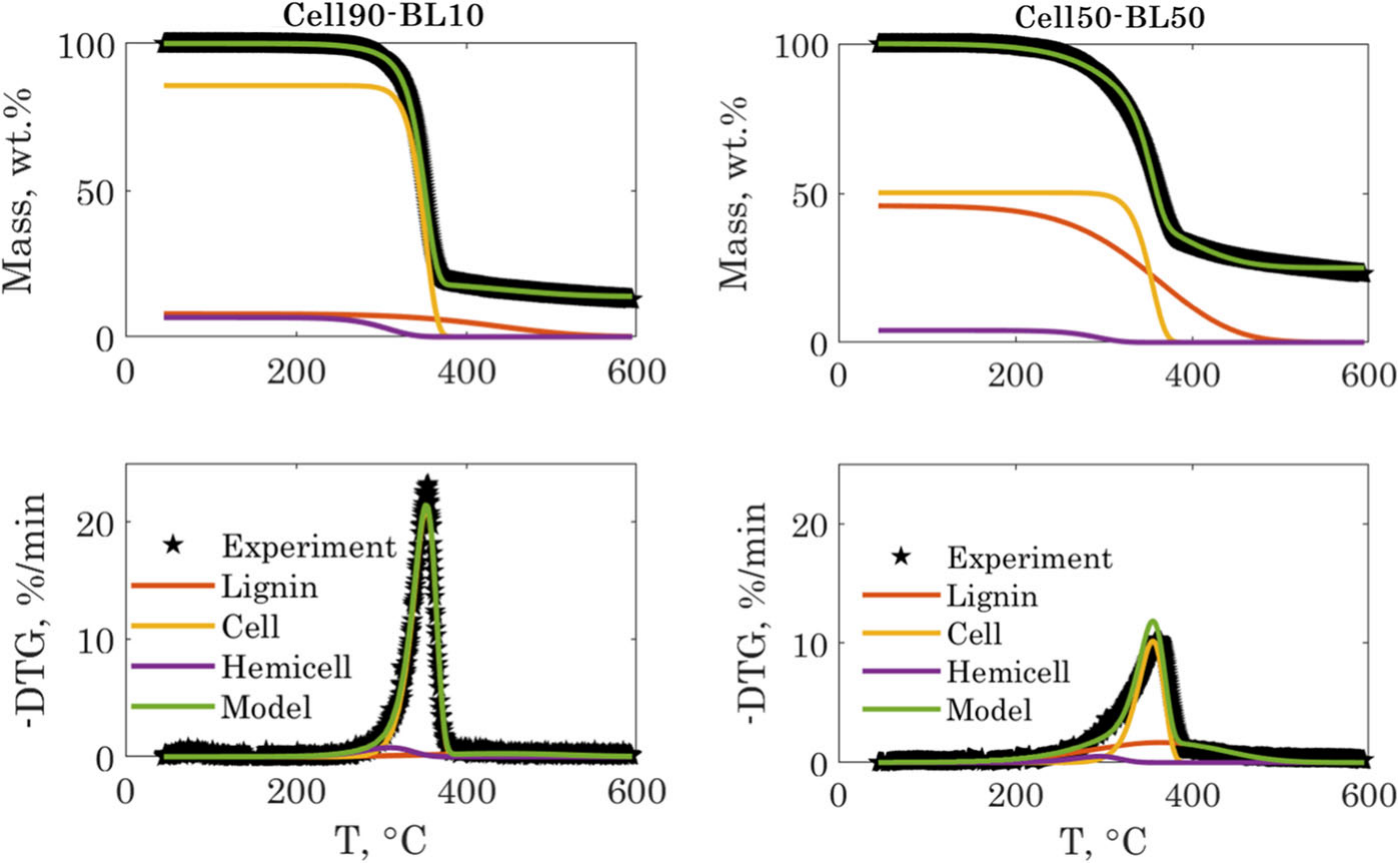
Experimental and modelling results for the E90-BL10 and E50-BL50 samples

The modelling results for the cases of Cell90-BL10 and Cell50-BL50 samples are shown in Fig. 4.

The identified kinetic parameters and the fit quality are given in Table 4. The reader can notice that the model captured well the TGA data for both cases. However, the values of the identified parameters for the two samples were different.

**Table 4:** Identified pyrolysis model parameters for the E90-BL10 and E50-BL50 samples

Sample	log ($A_{1}$)	log ($A_{2}$)	log ($A_{3}$)	$E_{1}$, kJ/mol	$E_{2}$, kJ/mol	$E_{3}$, kJ/mol	$c_{1}$	$c_{2}$	$c_{3}$	Fit, %
Cell50-BL50	0.67	18.10	6.20	42.4	242.6	91.0	0.70	0.82	0.90	1.41
Cell90-BL10	0.63	17.87	6.11	49.5	239.4	94.4	0.70	0.89	0.90	0.53

*The indices 1, 2 and 3 correspond respectively to lignin, cellulose, and hemicelluloses

**Fig. 5:**
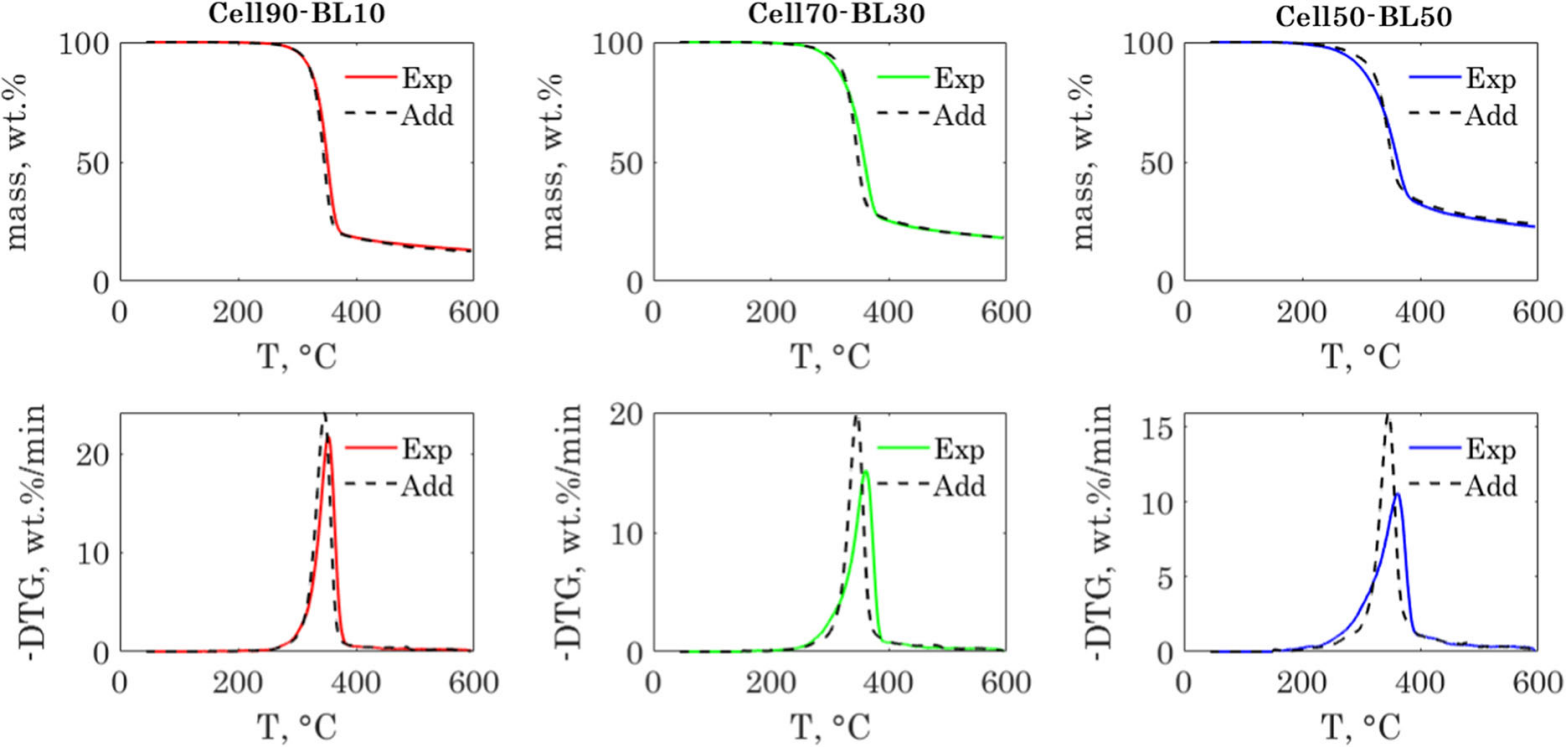
Comparison between the measured TGA and DTG curves of the cellulose-lignin hybrid fibers with the TGA and DTG curves (black dashed line) calculated following a superposition law

For instance, using the set of identified parameters for the Cell50-BL50 sample to model the thermogram of the Cell90-BL10 sample resulted into a Fit % of 4.01% instead of 0.53%, and into a Fit % of 2.2% and 6.09% respectively for the Cell70-BL30 and Cellulose samples. Deriving a single set of kinetic parameters which is applicable to all the samples will inevitably result in poor biopolymer content estimation. This is mainly due to the peak shift observed when increasing the lignin content in the hybrid fibers, which indicated a synergy between the closely packed cellulose and lignin during the pyrolysis reaction. A comparison between the measured hybrid fibers TGA and DTG curves and the calculated TGA and DTG curves following a superposition law is shown in Fig. 5. This figure illustrates well the difference between the hybrid fiber thermograms and those reconstrued using the superposition law. This difference becomes more pronounced when increasing the share of lignin in the fiber and explains why a model based on deconvolution or a superposition law fails to reproduce the original thermograms of the hybrid fibers.

**Fig. 6:**
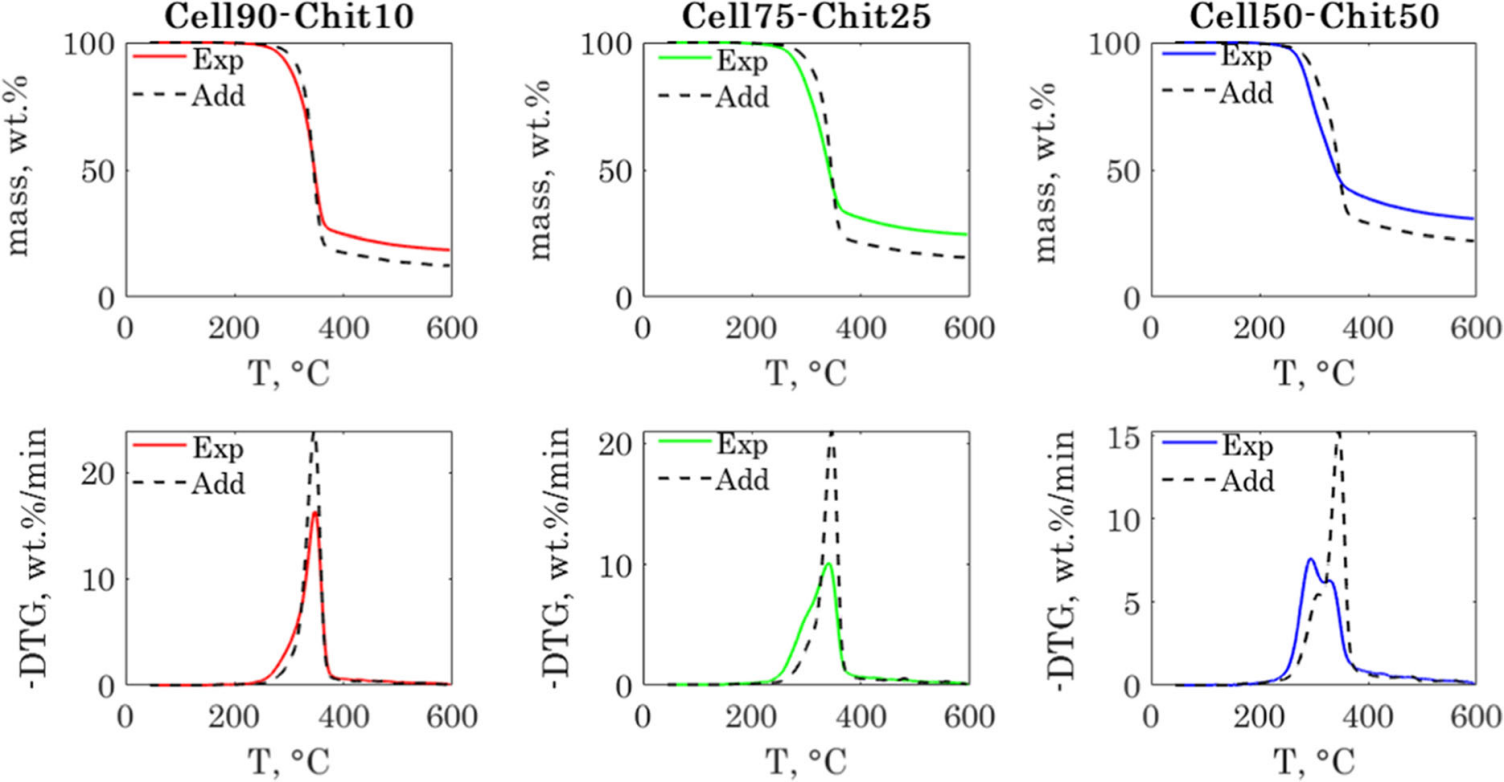
Comparison between the measured TGA and DTG curves of the cellulose-chitosan hybrid fibers with the TGA and DTG curves (black dashed line) calculated following a superposition law

The cellulose-chitosan case shown in Fig. 6 illustrates even more the limitations of the superposition law for predicting the composition of the hybrid fibers. The synergy between cellulose and chitosan during pyrolysis led to thermograms that were very different from the linear superposition cases. Moreover, it would be too complex and highly uncertain to come up with a pyrolysis model that describes those thermograms with sufficient accuracy and which can be used to identify the cellulose and chitosan shares in unknown samples. Clearly, the parallel reaction model cannot be used in this case.

To sum up, the synergy between the biopolymers during the pyrolysis reaction prevents using models based on superposition laws or models based on parallel pyrolysis reactions for predicting biopolymer contents in unknown fibers. In the presence of synergy between the biopolymers, the statistical modelling based on PLSR leads to more accurate predictions of the hybrid fibers composition.

## Discussion on the versatility of the TGA-PLSR thermoanalytical method

In this paper, we discussed the application of the TGA-PLSR method for analysing the composition of novel man-made regenerated fibers in which biopolymers were closely packed at the nanoscale level. This close packing leads to synergetic effects during the thermal decomposition, which make the prediction of a biopolymer content very challenging using classical modelling methods based on the linear superposition or on the deconvolution of thermograms. In that sense, the TGA-PLSR method is a more robust and reliable method for linking the information in the sample thermogram to its macromolecular composition.

The TGA-PLSR method can be applied to a wider class of composite materials in which the components show different thermal signatures. To illustrate our claim, we considered the example of textile blends composed of cellulose and polyethylene terephthalate (PET) fibers. Determining the composition of textile blends is important for the related industry since it is mandatory for textile producers to inform the customers about the composition of textile and garment products. The accepted tolerance for a fiber content is in the range of ± 2–5 wt.%.

Moreover, characterizing different textile fiber blends is crucial for determining the efficiency of emerging textile upcycling processes, where polyester fibers are quantitatively separated from cellulose fibers (Haslinger et al. 2019b). The classical wet chemical method for determining the cellulose or PET content in Cell-PET blends involves a harsh sulfuric acid treatment (ISO 1833–11: 2017) that depolymerizes and dissolves the cellulose chains, leaving the PET as a residue. The PET is afterwards washed, dried and weighed to calculate its share in the blend. The method is time consuming and involves the use of substantial amounts of inorganic acid and water. Researchers developed alternative methods for the compositional analysis of similar textile blends using near infrared spectroscopy coupled to multivariate PLSR (Ruckebusch et al. 2006; Mäkelä et al. 2020) or using solid state NMR (Haslinger et al. 2019a).

**Fig. 7:**
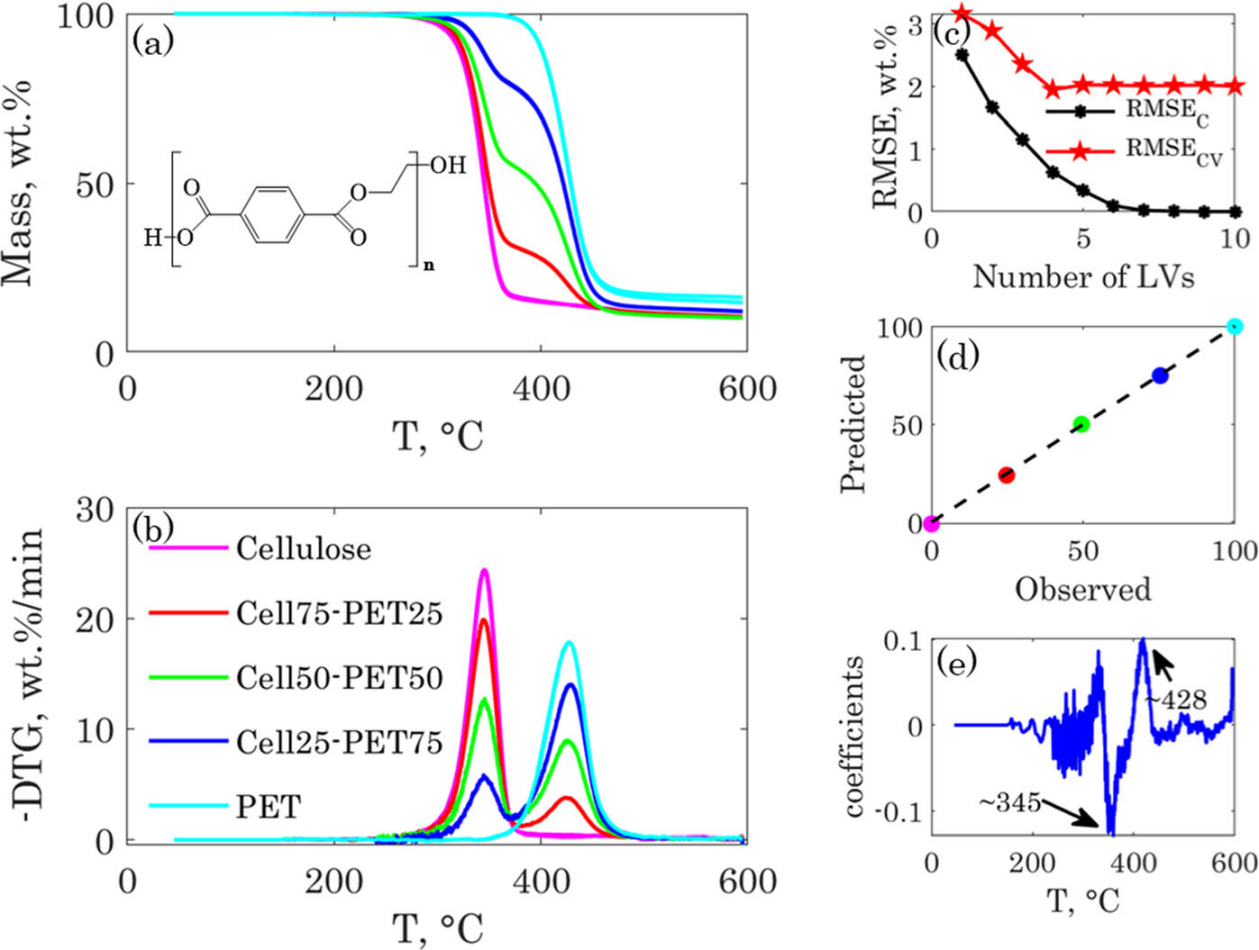
TGA (**a**) and DTG (**b**) curves of cellulose, PET, and their blends. The TGA-PLSR model RMSEs (**c**), predicted Vs observed PET content (**d**), and model regression vector (**e**)

The TGA-PLSR can also be applied to analyze the composition of Cell-PET blends. The thermograms of the Cell-PET blends as well as their derivative signals are shown in Fig. 7. The details of the experimental procedure and the characteristic thermograms parameters are given in the Sect. 5 of the ESI.

The thermograms preprocessing steps were the same as for the two previous cases and are illustrated in Fig.S13 in the ESI. The preprocessed spectra were used in the TGA-PLSR model for the determination of the PET content and the results are also shown in Fig. 7. Though the first LV captured more than 99% of the variance in the ***X*** and ***y*** blocks, we chose to integrate four LVs in the model as the ${RMSE}_{CV}$ showed a local minimum at four LVs. The scores and loading plots are shown in Fig. S14 in the ESI, and the reconstructed thermograms are shown in Fig. S15 in the ESI.

The cellulose and PET thermograms overlapped and were only partially resolved when blended. The TGA-PLSR method, however, fully resolved them by capturing their respective thermal signatures and generated a satisfactory calibration model with an ${RMSE}_{CV}$ of 1.94 wt.% and a coefficient of determination higher than 0.99. According to the standard method (ISO 1833–11: 2017), the confidence limits of the results obtained by the sulfuric acid method are not greater than ± 1 percentage point for the confidence level of 95%, which is in the same order of magnitude as the ${RMSE}_{CV}$ of the TGA-PLSR model.

Before we conclude this paper, we would like to add some final comments on possible sources of error that a diligent user of the TGA-PLSR method should be aware of. A list of recommendations is also given in the following section.

## Comments on possible source of errors and good experimental practices when using the TGA-PLSR method for the compositional analysis of composite materials

TGA-PLSR models can predict calibrated responses in unknown samples provided that the composition of the samples is within the model calibration range. However, a good prediction depends first and foremost on the quality of the predictor ***X*** block data supplied to the model. Therefore, users of the TGA-PLSR method should pay particular attention to external sources of errors that can affect a sample thermogram and hence lead to incorrect calibration and prediction of biopolymers content.

Experts on thermal analysis wrote extensively about external factors that can modify the thermogram (Brown 2005; Haines 2012). Those include, for example, the sample mass, the crucible geometry and material, the blank measurement, the gas flow rate, and the heating rate. These factors can cause temperature shifts in the thermogram or modify the sample mass loss rate. To avoid these unwanted effects, the user should design carefully the TGA protocol and stick to it during the calibration and the analysis of unknown samples. A non-exhaustive list of recommendations could be the following:

- The crucible material and geometry, the heating rate and the gas flow rate should be always the same during the calibration and the analysis of unknown samples.
- The sample mass and the heating rate should be low enough to minimize the influence of heat and mass transfer limitations. In this way, the mass loss dynamics will be controlled by the reaction kinetics.
- A blank measurement should be systematically performed when measuring a new set of samples, especially when a long period of time separates the calibration and the prediction stages. This blank measurement will correct any variation in mass which is not due to the sample thermal decomposition.
- A reference sample could be measured in parallel with unknown samples and hence serve to check for any change in the measurement conditions.
- It would be preferable to operate in inert atmospheres and avoid the use of reacting gases (e.g. oxygen when using air) because heterogenous reactions (exothermic combustion in this case) are much more subtle and difficult to control. Slight changes in the experimental conditions (e.g., changing the sample mass) can have significant effects on the thermogram.
- When predicting the composition of a new sample, the thermogram pre-processing steps should be the same as in the model calibration.

## Conclusion

We have shown in this paper that thermogravimetry and partial least square regression (TGA-PLSR) make a powerful combination for the analysis of biopolymers share in novel man-made cellulose-lignin and cellulose-chitosan hybrid fibers. No chemicals are used in this thermoanalytical method, the analytical time is relatively short (about 1 h), and the accuracy is good (cross validation error lower than 2 wt.% for lignin and chitosan contents in the range of 0–46 wt.%).

The present study also suggests that the TGA-PLSR method could be applied to a wider range of composite materials, as far as their components can be thermally resolved. We illustrated this versality by applying the TGA-PLSR method for the analysis of PET content in cellulose-PET fiber blends. The model results were as good as for the regenerated hybrid fibers. The method could predict the PET content in the cellulose-polyester fiber blends with a cross validation error of 1.94 wt.% in the range of 0–100 wt.%.

A comparison of the modelling methods that can be used for extracting compositional information from the thermograms of composite materials showed that the TGA-PLSR method outperforms more common modeling methods, which are based on linear superposition of reference thermograms or on thermogram deconvolution. The application of the TGA-PLSR method is particularly successful in the case of synergetic interactions between the (bio)polymers during their thermal decomposition. In such cases, the TGA-PLSR model can capture non-linear effects due to biopolymers synergism during pyrolysis, and leads to better predictions of their content than the two other modeling methods.

## Supplementary Information

Below is the link to the electronic supplementary material.

Supplementary file1 (DOCX 903 kb)
