# Supplementary material for: Fast and quantitative compositional analysis of hybrid cellulose-based regenerated fibers using thermogravimetric analysis and chemometrics
Source: Cellulose (Lond). 2021 May 28;28(11):6797–812. doi: 10.1007/s10570-021-03923-6 (PMC8550718; doi:10.1007/s10570-021-03923-6)
Supplement: Supplementary file 1 — Supplementary file1 (DOCX 903 kb) [file 10570_2021_3923_MOESM1_ESM.docx]

**Electronic Supplementary Information**

Fast and quantitative compositional analysis of hybrid cellulose-based regenerated fibers using thermogravimetric analysis and chemometrics

Authors: Chamseddine Guizani, Mikaela Trogen, Hilda Zahra, Leena Pitkänen, Kaniz Moriam, Marja Rissanen, Mikko Mäkelä, Herbert Sixta, Michael Hummel.

1. **Estimation of the thermal lag in the STA device**

An illustration of the STA furnace and crucibles holder geometry is given in **Fig. S1**. There are two crucibles on the sample holder: the reference crucible (R) and the sample crucible (S) containing the cellulose fibers.


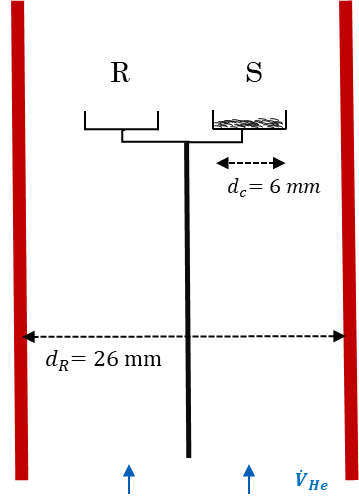


**Fig. S1**: Schematic illustration of the STA furnace and crucibles holder geometry.

Due to external heat transfer limitations, sensible and reaction heat requirements, the environment temperature $T_{e}$ (measured at the reference crucible) and the sample temperature $T_{s}$ might differ. The difference between the two temperatures corresponds to the thermal lag: $\Delta T=T_{e}-T_{s}$.

The magnitude of the thermal lag depends, among other factors, on the sample mass and heat capacity, gas type and flow rate, heating rate and the reaction thermochemistry.

By coupling a thermal model and kinetic model through the source term, it is possible to estimate to the real sample temperature $T_{s}$, which is more appropriate for deriving characteristic temperatures from the thermogram or for performing kinetic analysis.

The cellulose pyrolysis model assumptions and the procedure for estimating the thermal lag are described below.

Cellulose pyrolysis model assumptions:

- The cellulose sample is considered as a thermally thin body with a homogeneous temperature $T_{s}$.
- The cellulose sample volume and area remain constant during pyrolysis.
- The cellulose pyrolysis is a first order reaction with respect to the mass of cellulose.

The first-order rate equation of the cellulose pyrolysis reaction reads:

$$\frac{d\alpha}{dt}=k \left( 1-\alpha\right)$$

$$\alpha=1-\frac{m(t)}{m(0)}$$

$\alpha$ is the conversion level and

$k$ is the reaction rate constant, which follows an Arrhenius law.

$$k={A e}^{-\frac{E}{R T_{s}}}$$

Assuming that heat is transferred to the sample mainly through convection and is used to supply the sensible and pyrolytic reaction heat demand, the energy balance on the cellulose sample reads:

$$h A_{r}\left( T_{e}-T_{s} \right)=m_{0} \left( 1-\alpha\right) C_{p}\frac{dT_{s}}{dt}+m_{0}\frac{d\alpha}{dt}\Delta H$$

$h$: heat transfer coefficient

$A_{r}$: sample surface area

$m_{0}$: initial mass

$C_{p}$: cellulose and char heat capacity

$\Delta H$: pyrolysis reaction enthalpy

In order to estimate the thermal lag, we did a simulation of the pyrolysis reaction using the system of differential equations described above and the set of parameters given in the table below, which are representative of our TGA experimental conditions.

| **Parameter** | **value** |
| --- | --- |
| $m_{0},$kg | ${5 10}^{-6}$ |
| $\rho$, kg/m^3^ | 550 |
| $C_{p}$ (J/Kg.K) | 1670 |
| $h,$W/m^2^.K | $\frac{2.25 {10}^{-3}}{A_{r}}$ |
| $\Delta H$, J/kg | 210000 |
| E, J/mol | 226000 |
| Log (A, s^-1^) | 38.3 |
| HR, K/min | 10 |

The simulation results shown in **Fig. S2** indicate that the maximum thermal lag $\Delta T$ under experimental conditions close to ours is about 2 K.


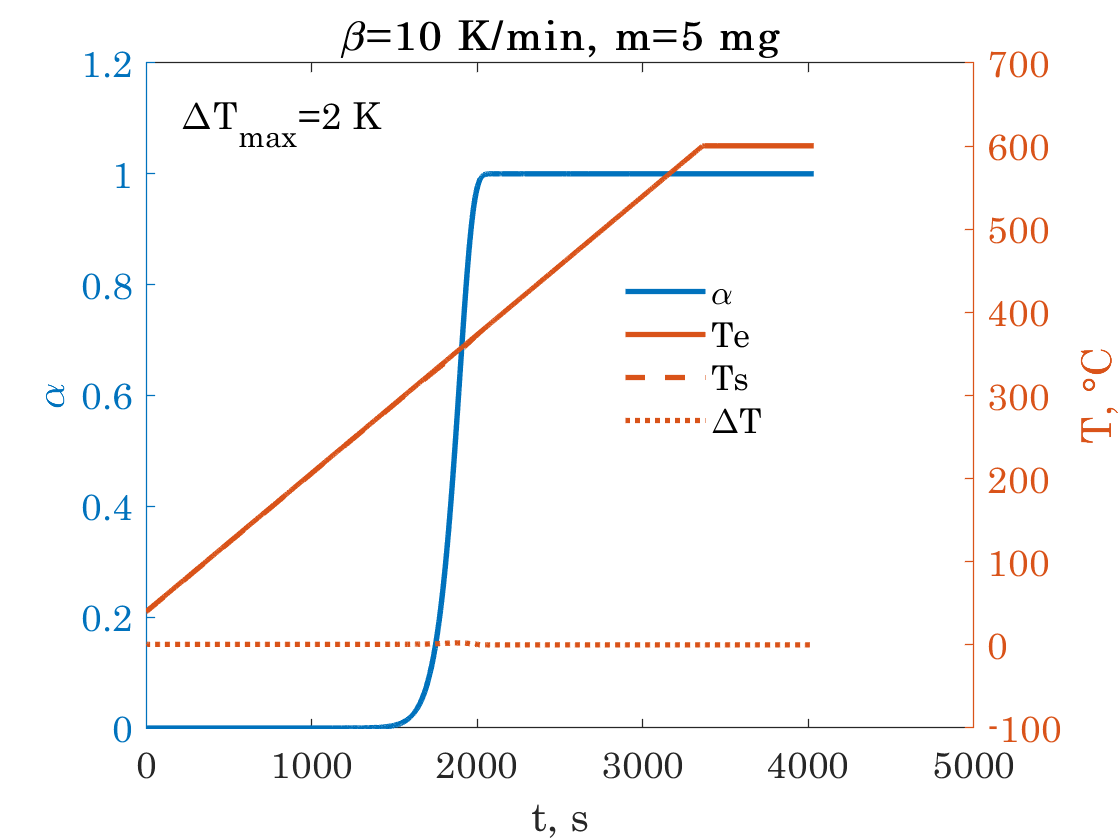


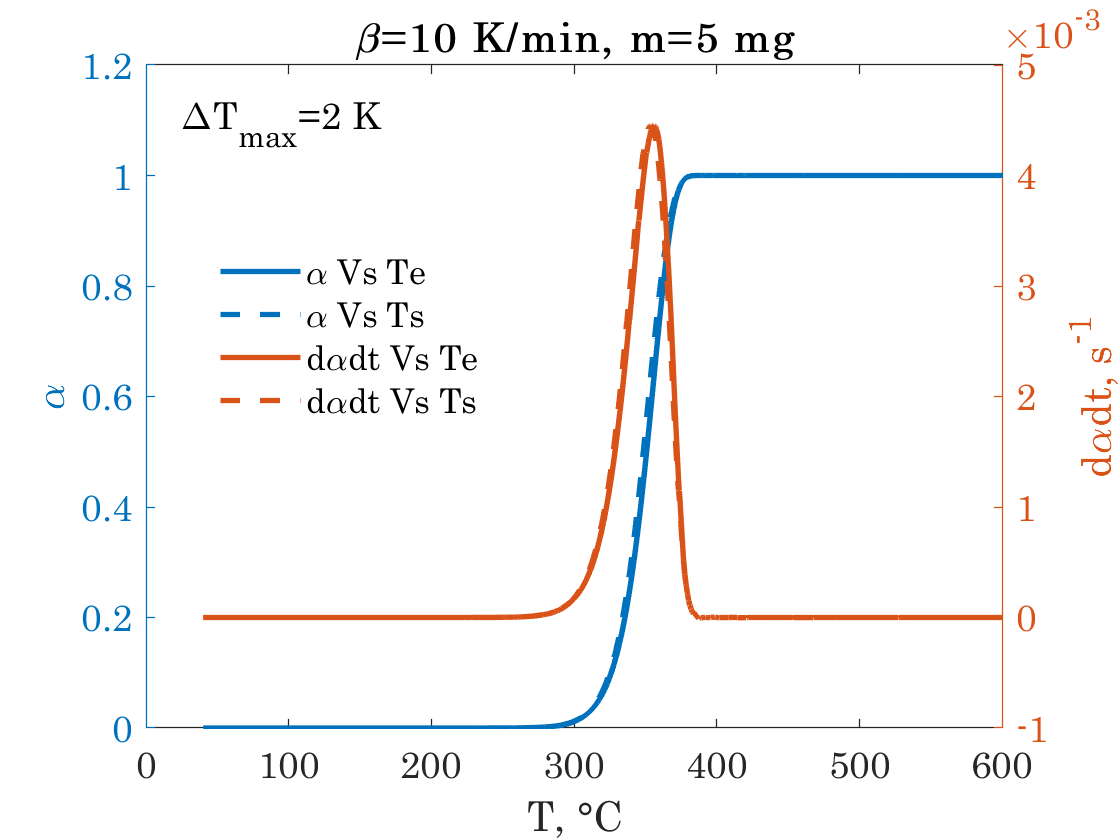


**Fig. S2:** Estimation of the thermal lag during cellulose fibers pyrolysis

1. **Compositional analysis of the cellulose-lignin and cellulose-chitosan fibers**

**Table S1**: Carbohydrates and lignin contents in the cellulose-lignin fibres.

| Sample | Cellulose, wt.% | ± | Hemicelluloses, wt.% | ± | Lignin, wt.% | ± |
| --- | --- | --- | --- | --- | --- | --- |
| Cellulose | 91.9 | 1.7 | 7.5 | 0.3 | 0.6 | 0.1 |
| Cell90-BL10 | 85.6 | 0.3 | 6.6 | 0.1 | 7.8 | 0.8 |
| Cell70-BL30 | 67.7 | 0.7 | 5.5 | 0.1 | 26.8 | 0.6 |
| Cell50-BL50 | 50.2 | 0.6 | 4.0 | 0.1 | 45.8 | 0.7 |

**Table S2**: Chitosan content in the cellulose-chitosan fibres.

| Sample | Chitosan, wt.% | ± |
| --- | --- | --- |
| Cellulose | 0 | - |
| Cell90-Ch10 | 7.95 | 0.16 |
| Cell75- Ch25 | 19.91 | 0.26 |
| Cell50-Ch50 | 44.06 | 0.58 |

1. **Thermogravimetric analysis**
   1. Repeatability tests for the cellulose-lignin samples


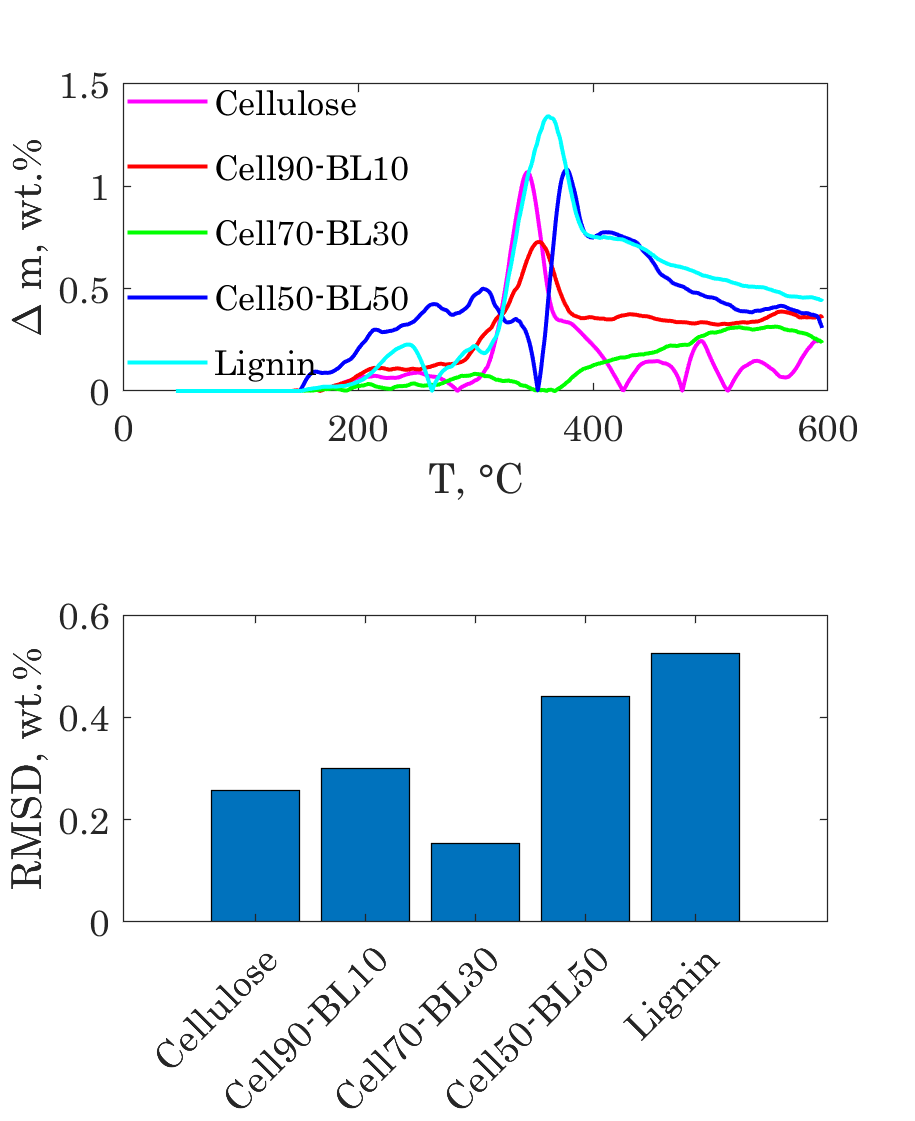


**Fig. S3**: Differences between the TGA curves of two repeatability tests (top) and derived root mean squared differences (bottom) for the cellulose-lignin samples

- 1. Correlation between the TGA characteristic parameters and the lignin content in the cellulose lignin samples


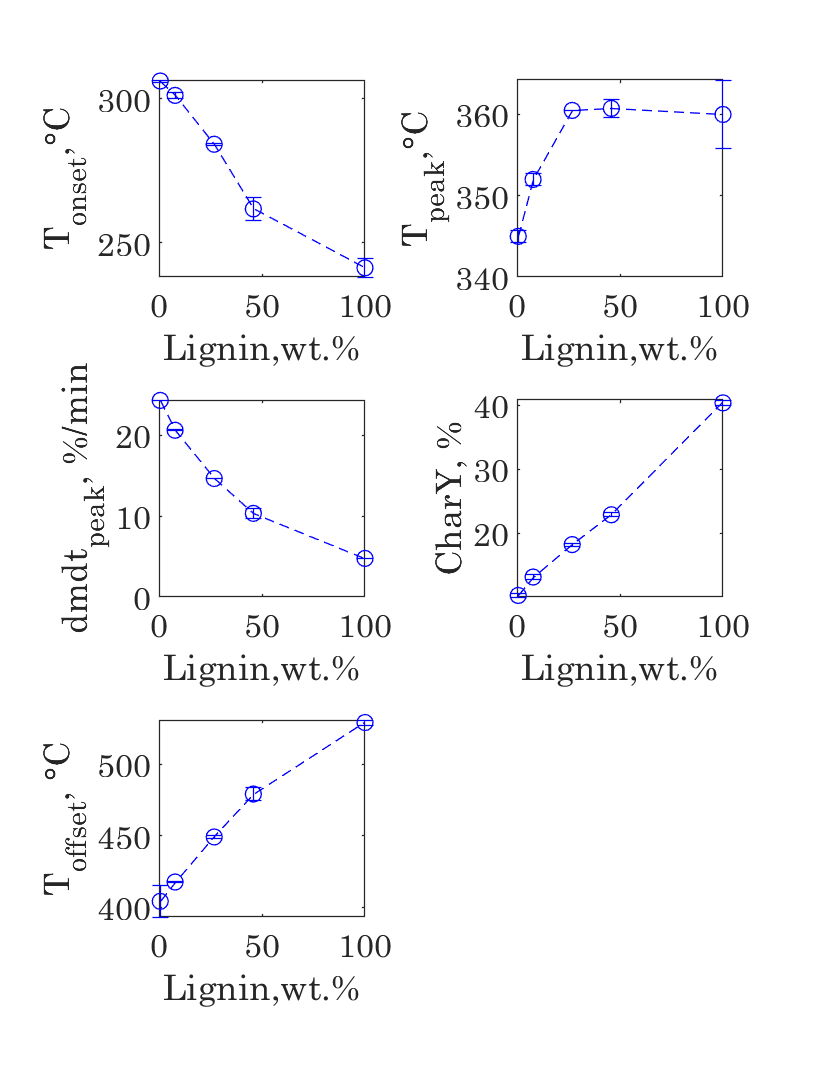


**Fig. S4**: Plots of the TGA characteristic parameters as a function of the lignin content (cellulose-lignin samples)

- 1. Repeatability tests for the cellulose-chitosan samples


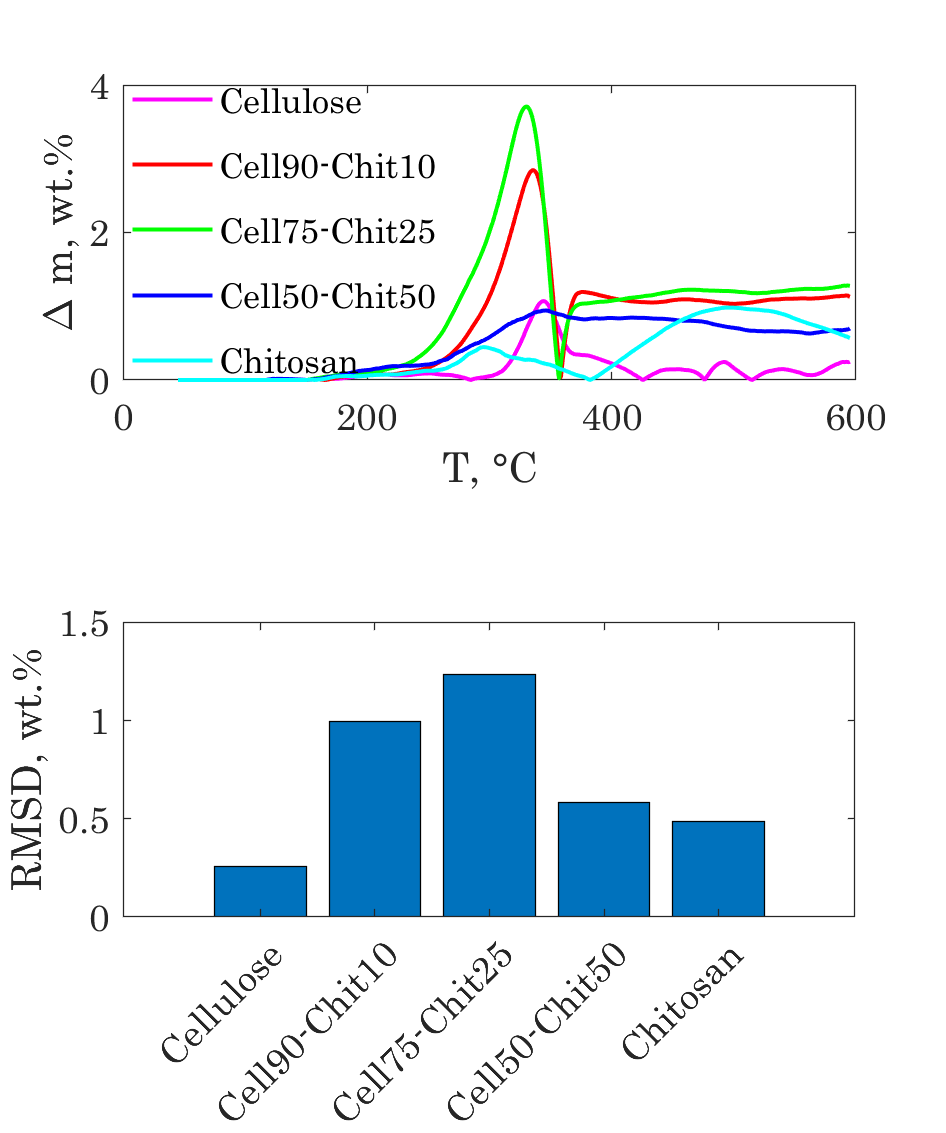


**Fig. S5**: Differences between the TGA curves of two repeatability tests and derived root mean squared differences (cellulose-chitosan)

- 1. Correlation between the TGA characteristic parameters and the chitosan content in the cellulose-chitosan samples


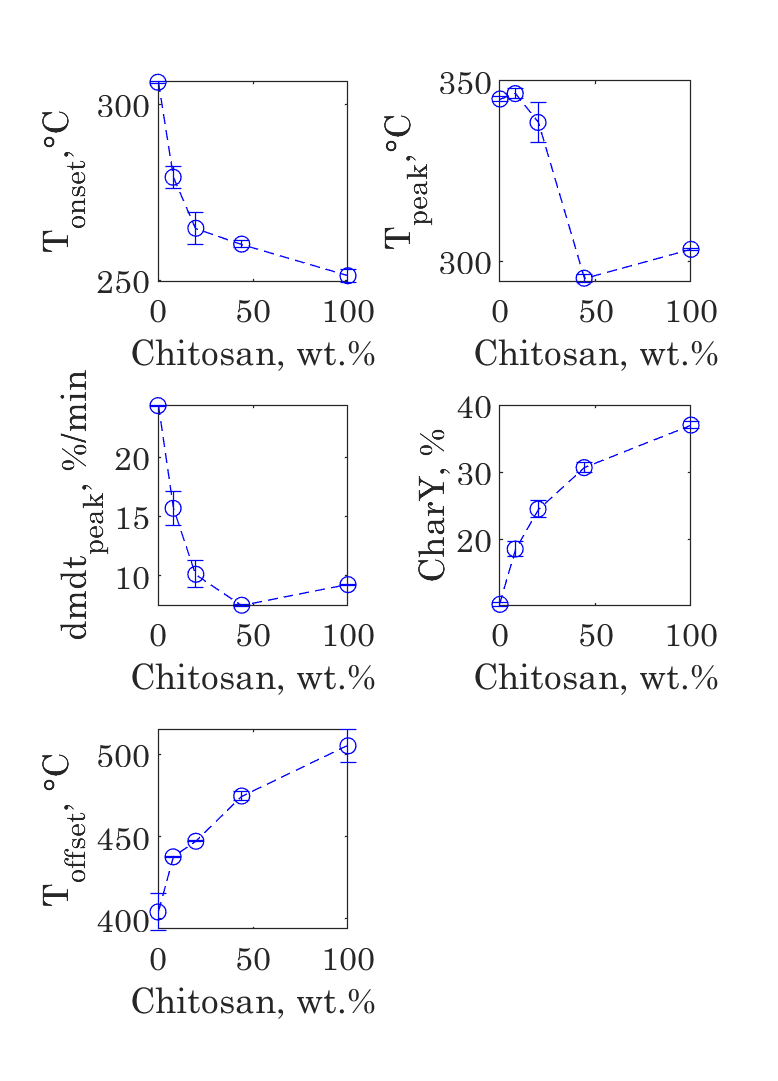


**Fig. S6**: Plots of the TGA characteristic parameters as a function of the chitosan content (cellulose-chitosan samples)

1. **TGA-PLSR modeling**
   1. Cellulose-lignin samples


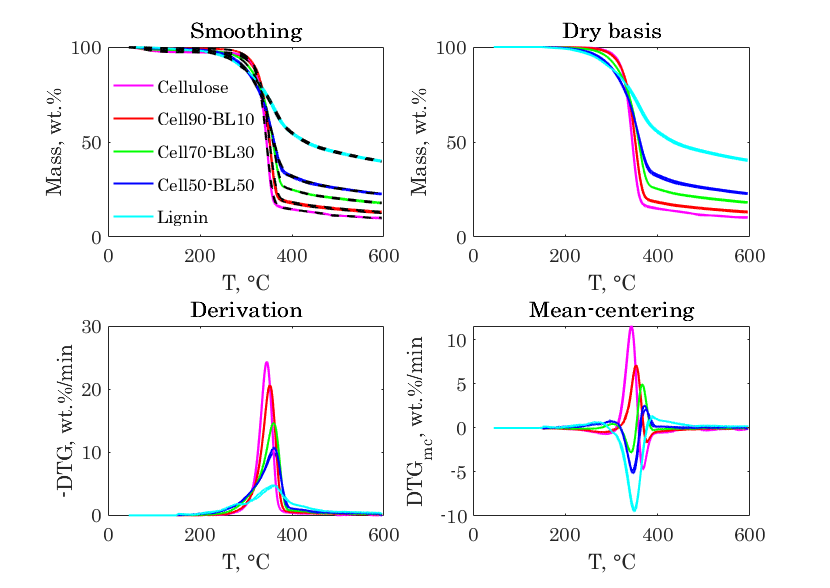


**Fig. S7**: Pre-processing steps of the cellulose-lignin samples thermograms


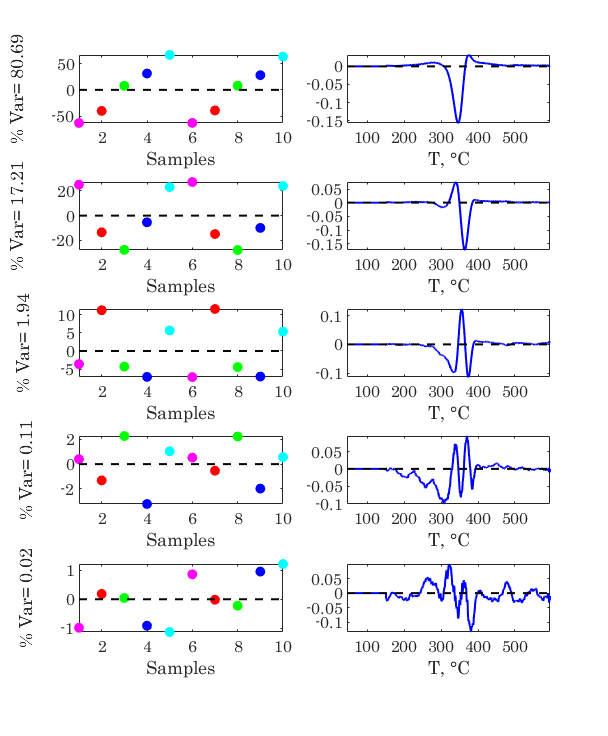


**Fig. S8**: Scores and loading plots in the TGA-PLSR model predicting the lignin content in the cellulose-lignin samples


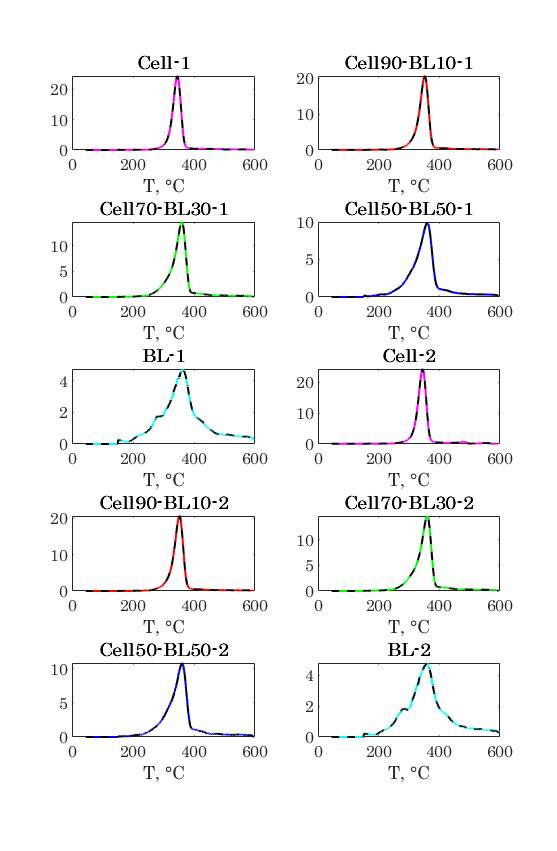


**Fig. S9**: Experimental (color) and modeled (black dashed lines) DTG of the cellulose-lignin samples

- 1. Cellulose-chitosan samples


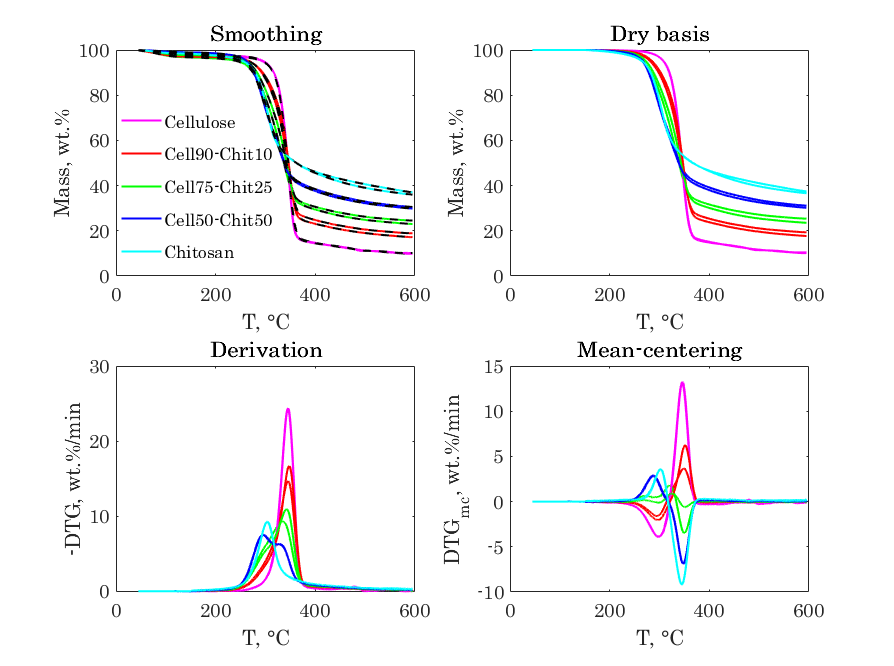


**Fig. S9**: Pre-processing steps of the cellulose-chitosan samples thermograms


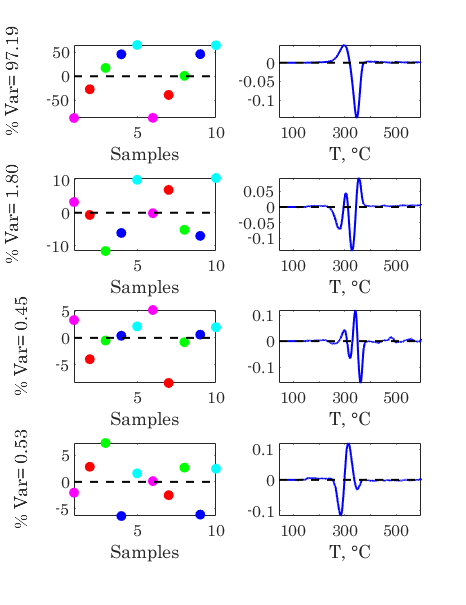


**Fig. S10**: Scores and loading plots in the PLSR model predicting the chitosan content in cellulose-chitosan fibers


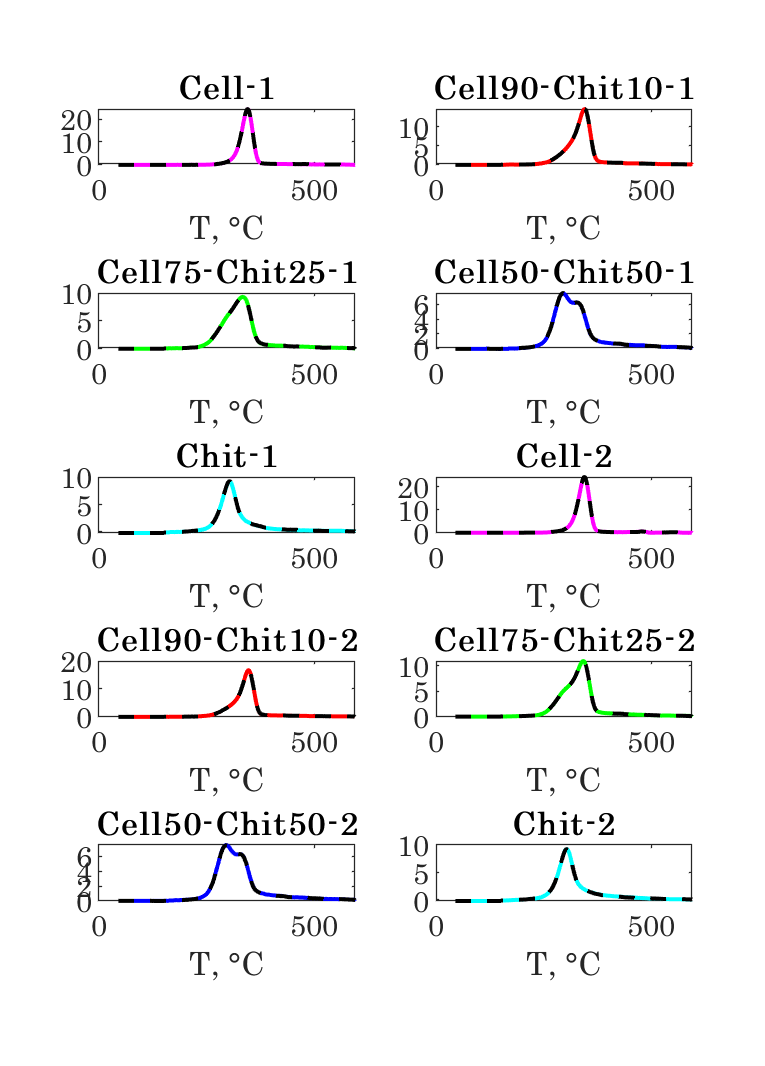


**Fig.S11**: Experimental (color) and modeled (black dashed lines) DTG of the cellulose-chitosan fibers

1. **The cellulose-polyester blends**

The cellulose-PET blends were prepared by physically mixing the regenerated cellulose fibers with polyester fibers inside the TGA crucible using an electronic scale (precision of ± 0.1 mg). The different samples and their shares in cellulose and PET are shown in **Table S3.**

**Table S3:** Cellulose polyester blends (Cell-PET)

| Sample | **Cellulose, wt.%** | **PET, wt.%** |
| --- | --- | --- |
| Cellulose-1 | 100 | 0 |
| Cell75-PET25-1 | 75.2 | 24.8 |
| Cell50-PET50-1 | 50.5 | 49.5 |
| Cell25-PET75-1 | 24.6 | 75.4 |
| PET-1 | 0 | 100 |
| Cellulose-2 | 100 | 0 |
| Cell75-PET25-2 | 74.6 | 25.4 |
| Cell50-PET50-2 | 50.3 | 49.7 |
| Cell25-PET75-2 | 25.2 | 74.8 |
| PET-2 | 0 | 100 |


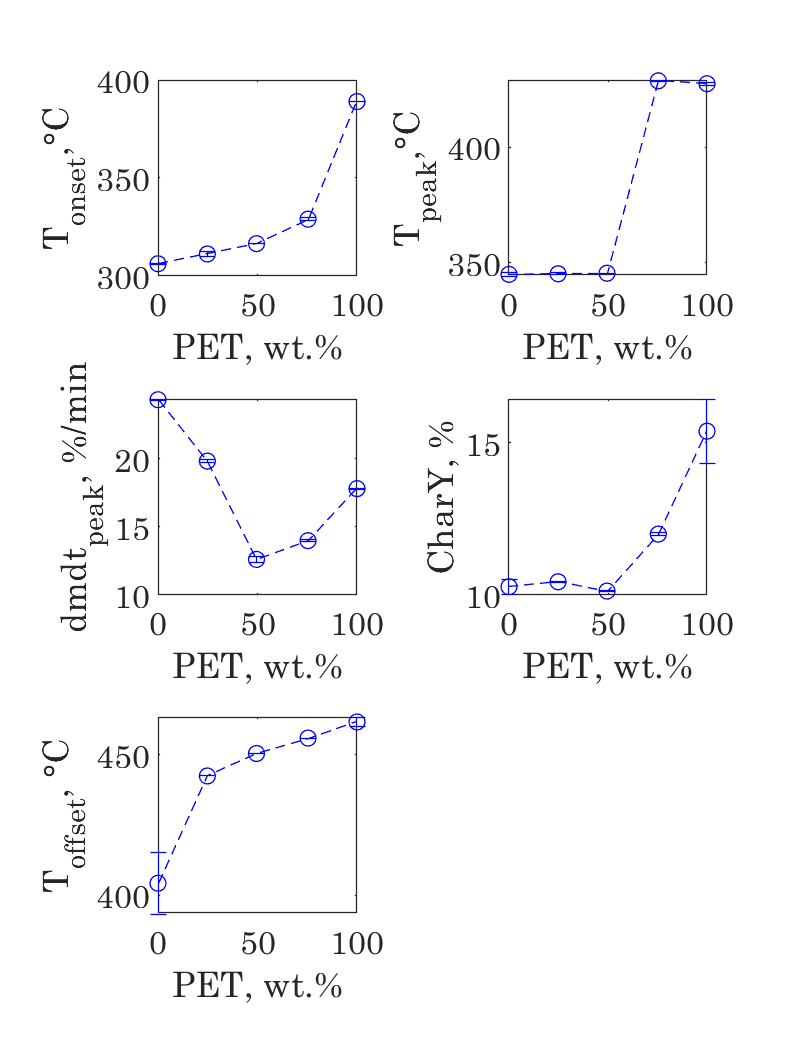


**Fig. S12**: Plots of the TGA characteristic parameters as a function of the PET content (cellulose-PET samples)

**
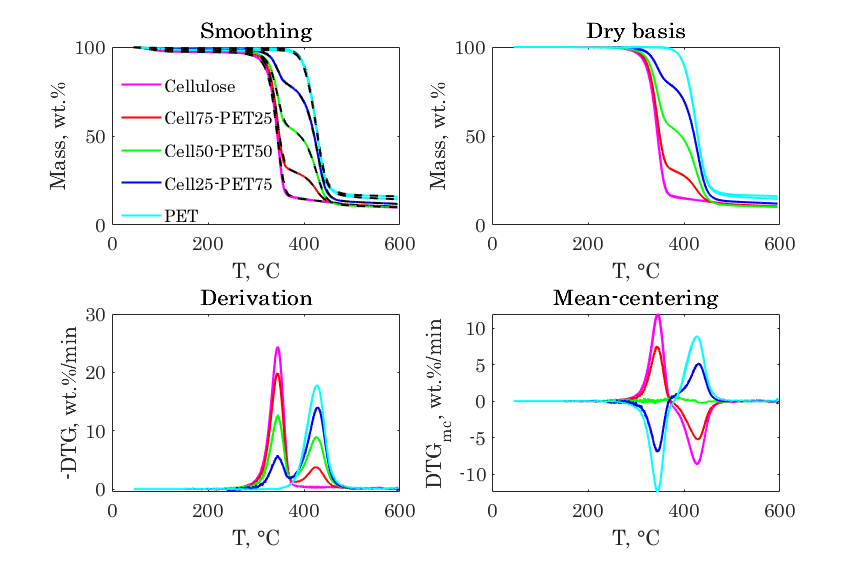
 Fig. S13**: Pre-processing steps of the cellulose-PET samples thermograms


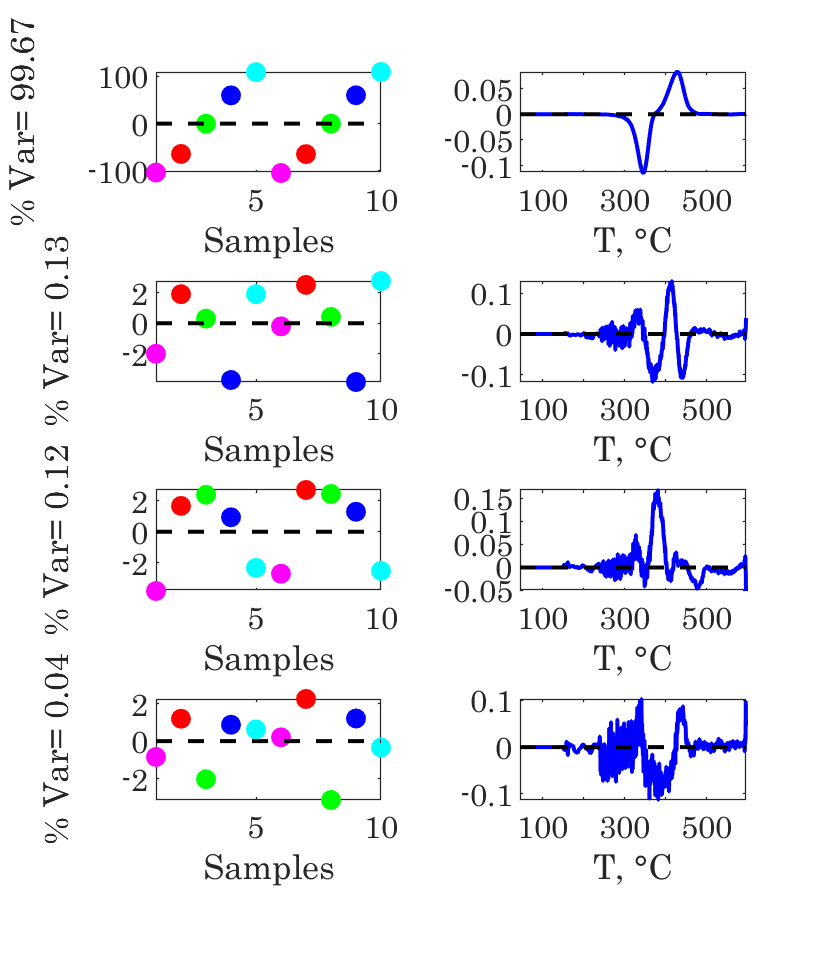


**Fig. S14**: Scores and loading plots in the PLSR model predicting the PET content in cellulose-PET blends

**
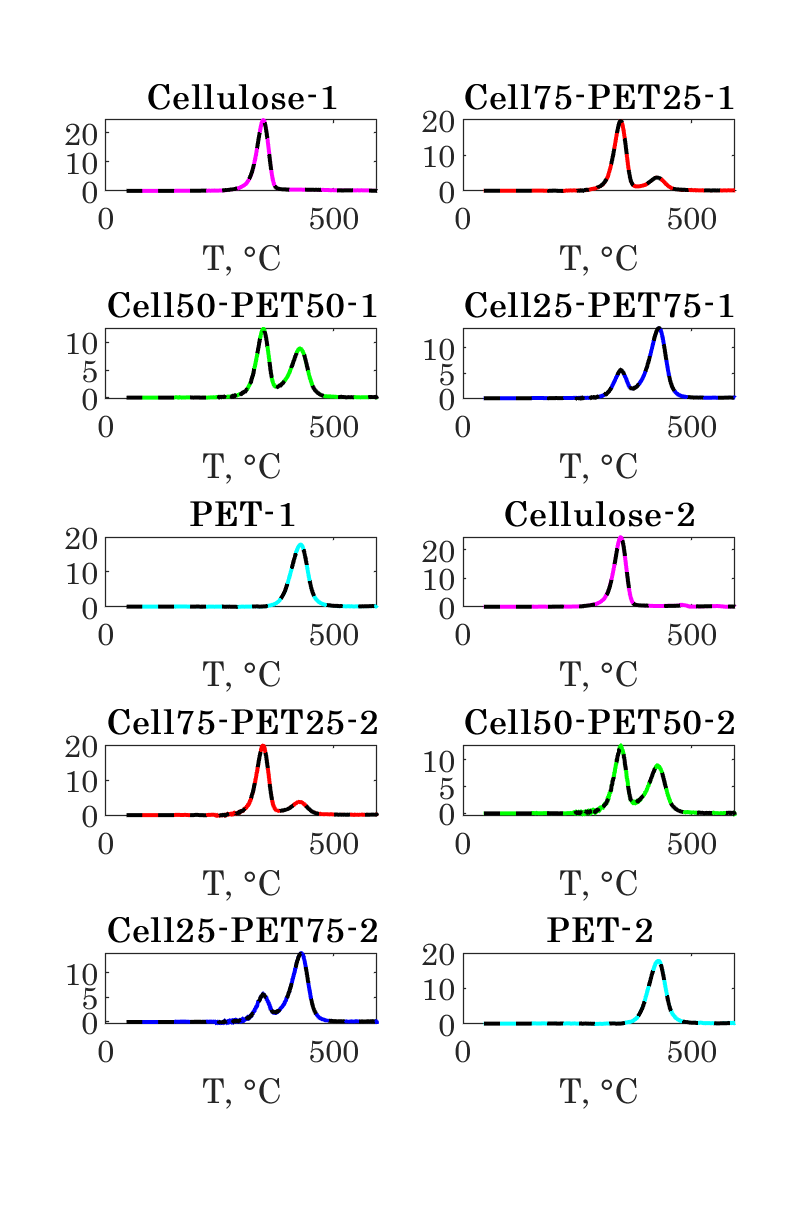
 Fig.S15**: Experimental (color) and modeled (black dashed lines) DTG of the cellulose-PET blends
